# An exploratory case study of food sharing practices in Caribbean countries through a transition lens using intergenerational dyad interviews

**DOI:** 10.1186/s12992-024-01094-0

**Published:** 2024-12-24

**Authors:** Anna Brugulat-Panés, Louise Foley, Madhuvanti M. Murphy, Nigel Unwin, Cornelia Guell

**Affiliations:** 1 MRC Epidemiology Unit, University of Cambridge, Cambridge, UK; 2 George Alleyne Chronic Disease Research Centre, University of the West Indies, Bridgetown, Barbados; 3 European Centre for Environment & Human Health, University of Exeter Medical School, Penryn, UK

**Keywords:** Caribbean Small Island Developing States, Nutrition Transition, Foodscapes, Food sharing, Intergenerational, Qualitative research, Non-communicable diseases, Well-being, Food and Cultural Identity, Globalisation

## Abstract

**Background:**

Caribbean populations face complex health issues related to diet and food security as they undergo a rapid nutrition transition, resulting in some of the world’s highest number of premature deaths from noncommunicable diseases (NCDs). Despite policy efforts to promote local and regional food consumption, reliance on food imports remains high with many Caribbean countries importing more than 80% of their food from larger economies. Previous regional research revealed the importance of food sharing practices in the Caribbean, with implications for the consumption of local foods, food security, and community resilience against climate change. However, there is limited understanding of these practices and how they have evolved.

**Methods:**

Using a transition lens, we explored the generational, life course, and sociocultural factors influencing food sharing practices in the Caribbean. We conducted semistructured online interviews with 10 intergenerational dyads involved in food sharing recruited from the virtual campus of the University of West Indies. Our study sample included 20 participants, ranging from 18 to 83 years old, from five English-speaking Caribbean countries (6 different islands).

**Results:**

Food sharing practices had a central role within the social fabric of all participants, contributing to their mental health and well-being. They represented a fundamental aspect of participants’ culture and traditions, driving a sense of belonging and connection among Caribbean communities. However, contemporary food sharing practices indicated a move towards more convenience-oriented and processed foods, with reduced time spent cooking and a decline in the use of food sources such as backyard gardens, fishing, and marketplaces, with a preference for supermarkets. These trends, particularly observed among younger generations, aligned with the ongoing nutrition transition in the Caribbean and were influenced by various sociopolitical and environmental factors such as climate events, economic fluctuations, urbanisation, and changing family structures.

**Conclusions:**

The evolving landscape of food sharing practices in the Caribbean has linkages with various domains of nutrition, health, society, culture, environment and globalisation. While a transition towards less healthy diets will increase the risk of many NCDs, the intrinsic cultural, social, and emotional benefits of food sharing could also positively impact health outcomes in the Caribbean region.

**Supplementary Information:**

The online version contains supplementary material available at 10.1186/s12992-024-01094-0.

## Background

English-speaking Caribbean populations are spread across 21 countries and territories [1], with a total population of approximately 8 million [2]. They face complex health issues related to diet and food security as they undergo a rapid nutrition transition [3, 4]. As described by Popkin in the early 1990s [5], this transition involves a shift from traditional diets rich in locally sourced fruits, vegetables, and grains to diets increasingly dominated by imported processed foods, high in refined sugars, unhealthy fats, and animal products. Often accompanied by rising rates of obesity, type 2 diabetes, and cardiovascular diseases, this shift reflects broader socioeconomic changes and globalisation influences on local food systems and consumption patterns [6]. While childhood stunting may not be prevalent in many English-speaking Caribbean countries, it remains an issue in some countries in the region, as does widespread micronutrient deficiencies. This combination causes a triple burden of malnutrition in the region — and a double burden in English-speaking Caribbean countries — leading to some of the world’s highest rates of premature deaths from noncommunicable diseases (NCDs) [7]. Despite important variations across the Caribbean countries in terms of their socioeconomic levels, histories, and political economies [8], these health challenges are a common concern. Some of the major factors driving these shared issues are geophysical and structural constraints, climate change, profound changes in food systems associated with globalisation and market liberalisation agreements, and inadequate local agriculture systems shaped by legacies of colonialism [8–11].

Many of the agricultural and economic models in the Caribbean have historically prioritised the cultivation of a few export-oriented monocrops. During the sixteenth century, European colonialism enforced plantation economies in many Caribbean countries, affecting their current agricultural practices [12, 13]. This enforced model created informal, unclear and complex arrangements for land access and use in the region [12], subjecting countries to societal class divisions that persist today, with some communities feeling little control over the food they grow and the land it is grown on [13]. As an example, Jamaica’s current food system has been profoundly shaped by the long history of the British colonial plantation economies, which established large-scale sugarcane production at the expense of local food production [10]. More recent historical contexts in this setting include Structural Adjustment Programmes from the 1970s, which have led to deregulation and market liberalisation [10]. These policies, also experienced across other Caribbean countries, have further weakened local food systems at the expense of food imports and specialisation in cash crops for exportation rather than for local consumption [14]. This prolonged emphasis on monoculture farming coupled with trade agreements, has resulted in underinvestment in domestic markets, such as reduced subsidies for farmers. Consequently, many food producers operate at a semisubsistence level, leading to a heavy dependence on expensive external markets [15]. Between 1990 and 2011, English-speaking Caribbean countries increasingly relied on food imports, with many of them importing more than 80% of their food from larger economies [16]. Some consequences of this growing dependency include challenges for local food producers in competing with the lower prices of imported processed foods and satisfying consumers’ shifting demands towards a greater variety of global foods and fewer seasonal restrictions [11]. To improve food sovereignty in the Caribbean, a key approach is increasing local food production and consumption, particularly for highly imported items like poultry, rice, and vegetables. In line with this goal, a recent policy, committed to by the heads of government of CARICOM (Caribbean Community) countries, aims to reduce the region’s food import bill by 25% by 2025 [17]. However, despite these policy efforts to improve food sovereignty in the region, progress has been limited. This is due to challenges such as top-down approaches with minimal citizen participation, lack of strategies to mitigate climate change-related issues, and unequal access to the benefits of the policies, particularly by small-scale farmers and women, as highlighted in previous research [12, 18].

Therefore, understanding how people in the Caribbean interact with and navigate within their local food system is important for addressing nutritional and health-related challenges. Research exploring these interactions appeared in the mid-1990s using the term ‘foodscapes’, which studied social and spatial inequities in public health and food systems [19]. Later research broadened this concept to include cultural and experiential aspects of the interactions between food and people [20]. This body of research, which studies the spaces that shape how people access and consume food, aimed to address the complexity of food systems, often taking long-term perspectives and focusing on related public health, social justice, and sustainability issues [21]. Findings from a parallel study we conducted on foodscapes, which included English-speaking Caribbean countries, highlight a variety of food sources —here referring to the various ways people obtain their food— existing in this region. Examples of food sources include, among others, grocery stores and marketplaces, as well as sources beyond physical (built) food environments, such as wild foods, home-grown produce, and government food assistance programs. These diverse sources of food were found to fulfil important functions for the Caribbean population beyond nutrition and to have broader links with economy, culture, environment, and well-being.

This evidence revealed the importance of food sharing practices as a food source in the Caribbean. ‘Food sharing’ refers to the diverse ways people obtain food for consumption without monetary means. Examples include communal feasting, bartering and gifting of foods, as well as food remittances. These practices differ from those of food aid, which include food donations from different types of institutions to people in need. There are indications that food sharing could increase the consumption of more locally grown foods, promoting more sustainable and healthier diets and potentially reducing import dependency [22–25]. However, an example from Jamaica showed that food sharing also involved the bartering of less nutritious and imported foods such as rice, flour, sugar, and tinned fish [26]. Through food sharing, communities seemed to improve access to certain foods, strengthening food security (especially in times of crisis) while emphasising community relationships and solidarity. For example, evidence from Puerto Rico described how fisherman routinely separated part of their catch to share with family, friends, and neighbours in need [27]. While these practices highlight community resilience and mutual support, their impact on dietary health and import dependency is nuanced, as they could include both nutritious and less healthy foods, sourced locally and imported. Overall, food sharing practices could represent an opportunity to reduce dependence on food imports in the Caribbean while contributing to the long-term prevention of NCDs and community resilience against climate change and other shocks. A deeper understanding of these practices in the Caribbean and how they are changing over time is needed to assess their potential.

Considering the dynamic nature of food systems and their influences in the Caribbean, we used a transition lens —here referring to the way people respond to change over time [28, 29] — to explore the role of generational, life course and sociocultural factors in contemporary food sharing practices and experiences in the Caribbean. We operationalised this notion of transition by interviewing intergenerational dyads to understand the fundamentals of food sharing practices, including their potential role in health, economic, social, and environmental domains. We explored the meaning and cultural value of these practices, as well as the factors influencing them and how they may have differed between generations and over time.

## Methods

We report this study following the Consolidated criteria for reporting qualitative research (COREQ) guidelines [30]. The checklist is available in additional file [see Additional file 1]. Additionally, the study protocol and relevant study materials are included in another file, offering comprehensive details of the methodology used [see Additional file 2].

### Research design, participants, and settings

This was an explorative qualitative study (*n* = 20) to gain understanding of the role of generational, life course and sociocultural factors in contemporary food sharing practices and experiences in English-speaking Caribbean countries. We conducted online interviews with intergenerational dyads involved in food sharing in the Caribbean. Using a semistructured interview guide, we aimed to obtain insights into the fundamentals of food sharing practices in people’s lives, their cultural value, and the factors that shaped food sharing over time and across generations. Using a dyadic form of interviewing, we aimed to gain greater insight from a transition perspective on how different generations experience the nutrition transition and based on this, how they approach food sharing practices differently.

We recruited participants through the University of West Indies (UWI) Open Campus. This is the UWI virtual campus with reach across different Caribbean islands, offering undergraduate and postgraduate programmes fully taught in English. We chose this platform for recruitment to ensure broad geographical representation, diverse socioeconomic backgrounds, ages, and genders and adequate internet access in this online study.

Eligible participants were residents in the Caribbean (see a list of eligible countries in Additional file 2), 18 years or older, had been involved in food sharing practices, and had an intergenerational partner (familial or other) to participate in the interview. Generally, we considered participants to be from different generations when they were born over a 15–20-year span, but we allowed shorter spans in three instances to obtain a better representation. Our study sample included 10 intergenerational dyads composed of 20 participants (17 female, 3 male) ranging from 18 to 83 years old living in the Cayman Islands, Grenada, Jamaica, Saint Lucia, and Trinidad and Tobago, who were interviewed between January and March 2023.

The participant characteristics are shown in Table 1.

**Table 1:** Characteristics of the participants included in the study

Setting	Relationship	Age ^1^	Gender ^1^	Age ^2^	Gender ^2^
Tobago	Granddaughter ^1^ - Grandfather ^2^	34	Female	83	Male
Trinidad	Mother ^1^ - Daughter ^2^	41	Female	22	Female
Saint Lucia	Mother ^1^ - Daughter ^2^	40	Female	18	Female
Cayman Islands	Father ^1^ - Son ^2^	49	Male	27	Male
Grenada	^*^Younger friend ^1^ - older friend ^2^	31	Female	41	Female
Trinidad	^*^ Younger friend ^1^ - older friend ^2^	55	Female	64	Female
Trinidad	Daughter ^1^ - Mother ^2^	27	Female	56	Female
Jamaica	^*^Older sister ^1^ - younger sister ^2^	29	Female	23	Female
Jamaica	Niece ^1^ - Aunt ^2^	23	Female	48	Female
Tobago	Daughter ^1^ - Mother ^2^	28	Female	**	Female

^1^ Dyad 1: Participants who were recruited through the UWI Open Campus and who identified Dyad 2 for the interview

^2^ Dyad 2: Participants were recruited by Dyad 1 and were not necessarily enrolled in the UWI Open Campus

*Instances where a shorter year span between participants was allowed

**Age not reported

### Data collection

Dyadic qualitative interviewing involves two people, who have an existing relationship, who interact with each other in a conversation guided by open-ended research questions [31]. This method allows each participant to develop personal narratives about a research topic while stimulating new ideas that might otherwise be missed [32]. An investigator of this study (A.B-P.) conducted the interviews online from the UK using the video teleconferencing software Zoom. Each interview lasted between 60 and 80 min and followed the pilot tested topic guide included in Additional file 2. The interviews were audio-recorded, and reflective field notes were taken immediately after each interview.

### Data analysis

We used NVivo Qualitative Data Analysis Software for analysis following a reflexive thematic analysis method. While our transition lens influenced the questions we posed to participants, we remained open to unexpected experiences and perspectives during analysis. As such, themes were developed following an inductive-deductive approach using transcript data and the study topic guide. The grouping of codes by study objectives and by themes are provided in additional file [see Additional file 3]. A.B-P. led the analysis in close discussions with L.F. and C.G. at each step and frequent consultation with M.M.M. and N.U. Interpretation of findings was discussed with all members of the team.

### Ethics

Local ethical approval for this study was obtained from the Open Campus Research Ethics Committee at the University West Indies (CREC-OC.0110/07/2022) and from the University Ethics Board of Psychology Research Ethics Committee (PREC) at the University of Cambridge (PRE.2020.087) [see Additional file 2]. All participants agreed to an electronic consent form and to have interviews audio recorded.

## Results and interpretation

Four key themes provided insight into how food sharing works and the functions it serves in the Caribbean settings we studied. The first theme investigated the role of food sharing in shaping the food culture and identity of the Caribbean. This is connected to the second theme, which examined the broader social aspects of food sharing. Our third theme explored how food sharing practices have evolved in response to nutritional changes in the region. Lastly, as we zoom out to a global perspective, we examined the contemporary global factors that influence the nature and extent of these practices.

### Taste of identity: Food sharing connections to Caribbean culture

Participants from Trinidad and Tobago referred to food sharing as an opportunity for ‘liming’. In this country, ‘liming’ is a colloquial term that dates back to at least the 1920s and describes the activity of hanging out while sharing food, drinks, conversation and laughter. The term is part of these islands’ culture, and it is believed to be used throughout the English-speaking Caribbean [33]. The fact that some participants made connections between food sharing and ‘liming’ symbolises the integral role of food sharing in shaping Caribbean cultural identities, heritage, and traditions.

#### The roots of food sharing

When we asked participants why they shared food, generally, the same type of response was given straight away: *“is something that I grew up with*,* it’s a norm. I would call it a norm of our country so probably from the same tradition that my grandpa grew up with from the poor times where they would share food with the neighbours and so on*,* so you grow up seeing that taken place so it’s just something that you continue to do.”* (34, female, Tobago). Thus, food sharing was something participants were raised with and described as something that *“it’s just part of you”* (40, female, Saint Lucia). It quickly became clear that sharing food was part of the participants’ identity, and as such, they were sharing food seamlessly: *“there’s nothing like a big heavy thought process about it. It’s just done. Yeah. It’s more of a natural instinct”* (27, male, Cayman Islands).

The participants reflected on how their food sharing practices could have originated. Some of them said that growing up in extended families had a strong cultural influence on the way they currently share food. Extended families are a type of family structure in the Caribbean where relatives live in the same neighbourhood or the same house and provide family support to each other, such as sharing meals and childcare responsibilities [34]. One participant said, *“you share with your neighbours because how the whole system of community living here*,* [.] most of our descendants would have come from like slavery and then we all lived in extended families [.]*,* so if one person has sweet potatoes and you and the other household has plantains and then the next one have green bananas*,* you would share with some what is it and then you just grow up doing it”* (41, female, Grenada).

Participants felt that their practices of sharing food were also influenced by their countries’ tradition of offering food as a sign of hospitality. They explained that preparing a little extra food was a customary practice, especially on a weekend, to ensure that if anybody happened to visit, they would have something to offer. This tradition of offering food or drinks seemed to go beyond just family and friends, and it also included people whom they were familiar with or even strangers. This mother said, *“Yeah*,* you never let people leave your home without eating or drinking something. That’s our culture”* (41, female, Trinidad), and her daughter added, *“You don’t let people leave your house empty handed.”* (22, female, Trinidad). The culture of hospitality extended to sharing food as a moral obligation, particularly when it involved people in need or aimed to prevent food waste: *“We may portion a bit and give it to them [colleagues or classmates] because having it for ourselves would be a bit selfish and say we get so much that it spoils on us*,* I think that would just not be right.”* (29, female, Jamaica).

Hence, participants attributed their engagement in food sharing to a blend of normative behaviours learned from childhood and ingrained in everyday life, coupled with a tradition originating in extended family structures and values of hospitality. These findings echo those of previous studies on Caribbean families, which emphasised the importance of family contact as a key factor in informal support exchanges [35–37] and provided new insights into the role of food sharing within these networks of support.

#### Caribbean cuisine in food sharing

Participants shared popular Caribbean dishes. These dishes included a wide range of adapted Indian, Asian, European, and Afro-Caribbean recipes showing the diverse influences that have shaped Caribbean cuisine over time. These recipes reflected the region’s history of cultural exchange and adaptation, as described by previous authors [38]. According to participants’ descriptions, these dishes were cooked following Caribbean culinary traditions where there is no room for fad diets or strict recipes. These dishes were cooked following one’s intuition rather than recipe books, cooking shows or any social media influencers. The participants seemed surprised when we asked them about recipes, “*Yeah. There’s no recipes. It’s all feeling*,* you know*,* just throw it in.*” (27, male, Cayman Islands). They got this ‘feeling’ for cooking from watching their elders in the kitchen or cooking together, as this undergraduate student explained to us, *“Oh no*,* we don’t have recipe books*,* we purely learn this from our parents*,* so when [.] our mum and dad cooked in the kitchen*,* we were in there with them*,* so we learn that from them*,* they show us the techniques*,* how to fry the chicken.”* (23, female, Jamaica). Consequently, food sharing practices seemed to play a role in passing down culinary knowledge through generations. These observations align with themes explored by previous authors in the fields of anthropology and human geography [39, 40] who delved into how culinary practices express cultural identities within the diverse historical and contemporary contexts of the Caribbean.

#### Food sharing, a unifying Caribbean tradition

Going beyond their personal experiences, we asked participants about their opinions on food sharing in other Caribbean islands. Some responses were shaped by their own trips to other islands and what they had witnessed. Others were based on stories they had heard from relatives and colleagues who travelled there. Some mentioned receiving information from TikTok and TV documentaries, while others referred to friends living abroad on various Caribbean islands. Despite the diverse sources of information, participants seemed to come to a common conclusion: *“food sharing is a Caribbean thing”* (41, female, Trinidad). Food sharing was seen as a unifying tradition that transcended geographical boundaries within the Caribbean region. This was attributed to the belief that Caribbean people had a family-oriented background ingrained in them, which led to food sharing. One participant did not have a formed opinion about food sharing in other Caribbean places, but she noted, *“I visited London*,* yeah*,* a couple of times*,* and the culture in the Caribbean is way different to the culture in London. So*,* like*,* for example*,* if I move into a new community*,* I would usually give my neighbour a cake*,* or my neighbour would give me a cake*,* or something like that*,* and we would talk and we would become friends. In London*,* it’s more like you keep to yourself*,* yeah*,* there’s no sharing of food [laughs].”* (27, female, Trinidad). Despite our study sample being from English-speaking Caribbean countries, participants drew connections between food sharing and the Caribbean culture (and made distinctions to other cultures), suggesting that these practices connect Caribbean people across islands and different colonial backgrounds, contributing to preserve their collective identity. Food sharing was seen as a source of pride, driving a sense of belonging and connection among Caribbean communities.

### More than meals: Food sharing as a nexus of social connection

In this theme, we examined the social function of food sharing practices beyond food and nutrition. We asked participants about the people with whom they shared food and how often. When asked about frequency, participants struggled to recall details, as they explained that while food sharing can occur on special occasions such as a family lunch on a Sunday, it can also occur sporadically on a daily basis without much thought. We found food sharing to be a widespread social activity, occurring not only on special occasions but also as part of daily life, involving family, friends, neighbours, colleagues, and even strangers. Some participants also shared food with people in need either on a personal level, such as by buying them a meal occasionally, or through organised community groups that distributed cooked meals or food hampers to vulnerable groups. For some participants, being part of a religious community and engaging in religious practices like prayer meetings or Christmas celebrations, also included the practice of sharing food with others. Despite the differences in their nature and frequency, food sharing had a central role within the social fabric of all participants.

#### Food sharing as an expression of care for others and for oneself

When sharing food, participants described feelings of love, care, and gratitude towards others as well as feelings of happiness and satisfaction with themselves. This manifested in different forms. This father explained, *“Sometimes we prepare meals for people specifically*,* and especially if they’re like mourning the loss of loved ones*,* we’ll prepare food for the grieving family and stuff. And we do it naturally. We just like bringing joy I guess.”* (49, male, Cayman Islands).

Other ways to show appreciation and love included adapting recipes to meet the needs and preferences of the recipients. This mother explained, *“When we season our food*,* we take into consideration that*,* okay*,* we maybe feeding young children*,* so we do not put you know*,* as much pepper in it*,* or maybe feeding older people who are*,* who have medical problems like high blood pressure*,* you know*,* these kind of things*,* so we try to cook with less salt and less pepper.”* (41, female, Trinidad). Younger participants also mentioned considering health when sharing food and looking after others. For instance, a young niece (23, female, Jamaica) shared fewer sweets with her younger cousins because she understood that they might not have the same level of responsibility for eating. This suggested that sharing food with others often involved being mindful of their well-being.

It became clear that such acts of care and generosity for others could also serve to support one’s own wellbeing. Some participants noted that cooking and eating were not the same unless it was shared: *“I get excited because I’m cooking for somebody*,* as opposed to if I have to cook for myself […] when I have to cook for someone else*,* I feel happy to do it so it helps with my mood and my wellbeing as well.”* (41, female, Grenada). This highlights how food sharing is related to positive emotions like joy and satisfaction in preparing and sharing meals with others.

#### Social bonding through food sharing

Food sharing practices served as opportunities for socialising. Participants talked about food sharing as an opportunity for spending more time together with family and friends, getting to know the neighbours, and building connections with others. *“It represents family time*,* it represents love*,* it represents friendship*,* closeness*,* a togetherness and unity*,* that’s what sharing food represents*,* it’s happy times”* (41, female, Grenada).

In this case, the food itself had a secondary role, and the main purpose was to facilitate the social space. The role of food sharing beyond the actual meals shared was also observed in special community events such as the ‘harvest’, a feasting to celebrate the harvest season, as this mother explained, *“you cook anything*,* […] people will just come and they will just eat*,* have fun*,* sit*,* talk*,* dance around*,* you know*,* and just enjoy themselves. You don’t have to be invited*,* you go from home to home*,* it doesn’t*,* on that day there is no*,* um*,* that said there is no enemy.”* (age not reported, female, Tobago).

These social activities linked to food sharing were seen as opportunities for family and community bonding, a way of making friends and creating long-lasting relationships, all contributing to people’s wellbeing. This was well illustrated by one participant who explained how they exchanged lunch boxes at work. This involved putting various foods in the microwave, selecting the dishes that each of them preferred, and making these arrangements before coming out for lunch together. Another participant added, *“At work*,* these are the people that you see mostly every day sometimes more than your family*,* […] we tend to know what’s going in everybody’s family or whose children is giving trouble or what problems we have*,* we tend to hash it over because when you share a meal it’s like you’re basically doing therapy at the same time”* (31, female, Grenada). Our findings align with previous research that emphasises the significance of social relationships in cultivating community bonding within Caribbean societies [41, 42] while also offer new insights into the role of food sharing practices in enhancing mental health and well-being.

#### Good manners in the practice of food sharing

Food sharing had its own ‘etiquette’, where questions about the origin, preparation, or purchase of the foods were generally avoided. This social rule helped maintain an atmosphere of respect and harmony within the community. However, even with these rules, some anecdotal negative experiences were unavoidable. For example, one participant shared a story of someone criticising their cooking skills, which led them to stop sharing with that person. This etiquette also applied to turning down food offered by others. The participants mentioned that younger generations tended to have somewhat more relaxed attitudes towards these conventions. Nowadays, it is generally accepted to refuse food for reasons like allergies, dietary restrictions, or feeling full. On the other hand, for older generations, not having a strong reason to refuse shared food could be seen as a sign of disrespect. This illustrates differing attitudes towards food sharing between age groups and reflects changes in the sociocultural meaning of food sharing practices over time. Overall, the fact that food sharing had a code of conduct, which evolved over time and adapted to sociocultural changes, reflected the importance of this practice within society and could have implications for nutrition, a topic we explore further in the next theme.

### Food sharing and the nutrition transition in the Caribbean

In this theme, we explored how food sharing practices evolved over time, illustrating their alignment with the characteristics of the nutrition transition in the Caribbean region. Participants explained how they share food nowadays, offering details on the types of foods exchanged and where they obtained it from. While our primary focus remained on food sharing, our discussions naturally extended to broader aspects of participants’ food habits, as these habits were relatively consistent whether or not they intended to share the food. As a result, our analysis of food sharing practices also provided insights into how people obtained, prepared, and consumed food for themselves.

#### Intergenerational differences in food sharing

While food sharing practices had many commonalities among all participants, some noticeable generational differences appeared. Typically, older participants were more used to buying ingredients from a variety of sources, including supermarkets and local markets, to make their own meals, which they would then share with relatives, colleagues, neighbours, or friends. At times, they would source ingredients from their own backyards, especially when it came to fresh fruits and herbs for seasoning the meals they cooked and shared. These dishes were often popular Caribbean dishes. Their recipes were varied and included marinating the meats overnight, preparing plantains and vegetables from scratch, and making blended drinks in large batches from fresh fruits and vegetables. In contrast, participants noticed that younger generations nowadays buy most of their ingredients from supermarkets, often opting for ready meals, soft drinks, snacks, or candies that they share with their friends. One participant said, *“You’re starting to see a difference in the food that is being shared across generations in a sense. So when we were growing up*,* […] we had what we call Independence day […] You used to bring your food to share or you would cook a food*,* whether it’s the national dish or whether it’s one friend bring a pie and you bring salad but now what we’re noticing as this generation*,* they tend to buy a lot of the fast foods”* (41, female, Grenada). When asked about intergenerational differences in their approaches to preparing shared foods, this mother gave the following example: *“I might use fresh seasoning*,* you know*,* I’ll pick the seasoning from my backyard*,* she [the daughter] might use the ground one already made in the supermarket in the bottles”* (41, female, Trinidad).

During a conversation between an aunt and her niece, we observed how intergenerational differences could transcend into the way younger individuals interact with and perceive certain food sources. They said,

*“ - (aunt): I go to the local market and every Saturday.*

*- (niece): I hate the market because it’s too crowded*,* and sometimes when you go*,* they profile you*,* so if you go and you look kind of a way*,* then we’re going to charge you enough money. And I won’t have it*,* I just go into the supermarket and avoid all of the robbers walking up and down and just pick them [foods] up.*

*[…]*

*- (aunt): I go to the seaside and get it [fish] from the fisherman as they come in from sea.*
*- (niece): I go to the supermarket [all laugh].”* (48 and 23, females, Jamaica).

As seen in previous evidence [43–45], marketplaces create social ties that generate familiarity, loyalty and commitment between vendors and customers. In this example, the niece did not experience any of these customer-friendly practices to cultivate loyalty, which affected her preference for supermarkets.

#### A shift towards a culture of convenience in food sharing

Changes in food sharing practices that mirrored the nutrition transition (from fresh, more home-grown, or home-prepared foods to processed, bought or preprepared meals; [3, 5, 6]) were not limited to different generations. They were also evident within the same generation when we looked at how they used to share food in the past compared to now. These changes over time were evident in how they sourced and prepared the foods they shared, now leaning towards a culture of convenience. The participants said this shift could be due to changes in their lifestyles and the resulting limited time availability, involving a shift towards purchasing food rather than growing it. As this mother put it, *“No*,* no*,* I work Monday to Friday then I go to school*,* I don’t want my Saturdays and Sundays to be planting at the back garden*,* I don’t want that.”* (40, female, Saint Lucia). Participants were also more inclined to buy food from supermarkets rather than from a variety of sources due to its convenience, and to spend less time cooking meals. This participant mentioned, *“In the week when I’m bringing lunch*,* I look about stuff that’s easier to cook*,* like maybe canned food*,* and then on the weekends*,* I make chicken*,* or one day in the week*,* I’ll make chicken and bring to work”* (23, female, Jamaica).

### Food sharing in a globalised world

As we explored changes in food sharing practices over time and across generations, we realised that these practices are not ‘hermetic’ or isolated from contemporary life. This theme delves into how food sharing practices are intertwined with global events and influences.

#### The effects of the pandemic on food sharing

COVID-19 also affected how participants shared food during that period. Some of these changes were still present, with certain effects yet to return to their prepandemic forms. Overall, participants felt that sharing food became more challenging during that period because they spent most of the time at home and felt cautious about going out and potentially exposing themselves and their families to the virus. As a result, they narrowed down the number of people they shared food with and how often they did it. However, within extended families, food sharing seemed to increase during the pandemic because all family members spent more time at home. A few participants noted that the pandemic made people go to supermarkets less frequently due to repeated lockdowns. This led participants to rely more on fruits from their own gardens or their neighbours.

#### The effects of climate change on food sharing

Food sharing was also impacted by changing weather conditions, which became more severe due to climate change. Participants expressed concerns about the damage caused to food crops by recent floods, the imposition of water restrictions due to inadequate rainfall during the rainy season, rapid crop drying due to high temperatures, increased frequency of hurricanes, and the decline of coral reefs. This father shared, *“We have a lot of coral bleaching and stuff and it’s because of high water temperatures and stuff*,* so we see reefs when I was a boy that was vibrant is now all dead. When I was growing up and my father was cooking beef or something and I didn’t want beef*,* I could just go to the reef and get a lobster and then come and cook. That whole reef is now dead. So it makes it difficult for my now grandchildren to have the same lifestyle that I enjoyed as a boy*,* and I think that [the] level of unsustainable living needs to be curved”* (49, male, Cayman Islands). In line with previous research [46, 47], participants felt that due to these events, the availability and accessibility of food became more uncertain and expensive. Participants were struggling to afford enough food to meet their family’s needs, and as a result, they were unable to share food as frequently. One participant explained, *“because of the flooding [.]*,* all the prices went up*,* so because the prices have gone up*,* it’s difficult now to supply for yourself and to share.”* (64, female, Trinidad). However, it was also noted that after extreme climate events, people tended to share more as an act of solidarity with others: *“you would share a bit more during the hurricane season too because maybe not everybody was able to stock up appropriately.”* (23, female, Jamaica).

#### Economic impact of global factors on food sharing

Participants discussed various sociopolitical and economic factors that currently shape their food sharing practices. As seen in previous research [48], the growing population in urban areas appeared to complicate food access for some participants, who tried to manage by relying on food remittances from relatives living in rural areas. These challenges resulted in a decreased ability to share surplus food, as described by one participant: *“Fishing was a whole lot easier because there was like only 20*,*000 people leaving here when I was a boy 30 years ago*,* now it’s 80*,*000 people living here*,* so even when I go to fish now I’m not guaranteed to catch ten fish anymore*,* you know*,* and if I need to feed my family of seven I need ten fish*,* [.]. I cannot have a fish to give.”* (49, male, Cayman Islands).

Every participant highlighted the significant increase in food prices they were experiencing on their respective islands. These price hikes occurred frequently, with costs doubling or even tripling within a matter of weeks, as reported by the participants and also by other authors [12, 46]. The causes behind these price increases were varied, with participants mentioning climate change along with COVID-19 and supply chain challenges. Regardless of the cause, the end result of high food prices was a direct impact on people’s purchasing power, consequently affecting their ability to share food: *“before we could buy 10 lb of rice and cook 10 lb of rice*,* and the price was pretty stable*,* but now you would find yourself buying 4 lbs [due to the high food prices] [and] because you also have to get to eat*,* it kind of limits on the amount that you can share.”* (41, female, Grenada).

Food sharing was often linked to socialising activities and as such increased rates of unemployment and crime were also identified by the participants as factors that impacted food sharing. This mother shared, *“when I was smaller sharing food was a lot more common then*,* you know*,* a lot of people they started to keep to themselves*,* […]*,* with the crime situation and all of these things now*,* you know*,* people keeping to themselves a lot more.”* (41, female, Trinidad). This sentiment was echoed by her daughter, who explained that, due to the rising crime rate, *“you usually don’t know who to trust now*,* you keep to yourself […]*,* you don’t tend to socialise with neighbours or people you see out on your own*,* you’d be a little more cautious now because of how common these occurrences have been coming”* (22, female, Trinidad). She also added: *“food is usually associated with socialising in our culture*,* [but we] can’t afford as much to go out and to socialise”* (22, female, Trinidad). As a result, participants became more selective in their food sharing, predominantly choosing to share only with their close-knit circle of family and trusted friends. In light of these findings, future research could benefit from adopting a systems-informed approach to better understand the complex interactions between the economy, the environment and food sharing practices in the Caribbean.

#### Perceptions from different generations on the decline of food sharing

Despite engaging in conversations about the global uncertainties and challenges that impacted their daily lives, the decline in food sharing was perceived as a negative thing, characterised as a manifestation of greed. A number of participants observed a shift towards more individualistic behaviours, which in turn influenced the practice of food sharing. An elderly participant shared this sentiment: *“People is all for themselves no matter how much you get. They don’t want to share”* (83, male, Tobago). This behaviour seemed more common among younger people; as one participant put it, “*I think some things have changed because people are now more into themselves*,* I think we are becoming more of a me-me-me generation*,* this younger generation is more into themselves*,* so they’re not as freely giving as the older generation everyone have.”* (64, female, Trinidad). However, younger participants defended themselves from these criticisms by mentioning the challenging times they had to live, as this one said, *“I don’t have a millionaire job to do that*,* so no I cannot always share food. […] It doesn’t make sense financially for me*,* because I sent myself to school*,* so it would be hard to buy food to give out”* (23, female, Jamaica).

After considering these ideas, it became clear that having a surplus of food played a crucial role in facilitating food sharing. As some participants pointed out, in the past, the act of food sharing was often driven by necessity. The absence of refrigeration meant that people had to share to prevent surplus food from going to waste. This awareness of minimising food waste had persisted over time, as this aunt said, “*admittedly if you don’t share sometimes you’re just going to have this leftover and then it’s just gone to waste*,* so why keep leftovers and let it to waste when you can share with somebody that need[s] it*” (48, female, Jamaica). However, during times when resources were scarce and obtaining enough resources for oneself was already challenging, there was a potential risk that the practice of food sharing may have been lost if nothing was done. A young man explained that in his generation, sharing with friends was common, but sharing with strangers was less common due to increased caution. He mentioned that his parents’ generation shared more freely with everyone but that his generation faced challenges in keeping this practice due to evolving circumstances. He expressed concern that the younger generation after his is even less inclined to share, and he concluded, *“It’s kind of up to my generation to be like the last battalion to kind of keep it [food sharing] together because if we don’t keep it together*,* then we’re going to lose it all. That’s how I see it. […] The current generation potentially losing that [food sharing] puts a big hamper on our culture as people too.”* (27, male, Cayman Islands).

## Conclusion

This study offers valuable insights into the evolving landscape of food sharing practices in the Caribbean, highlighting their connections to nutrition, health, society, culture, and the dynamics of our globalised world. Our study reveals that food sharing practices are deeply embedded in Caribbean culture, reflecting values of community, care, and social cohesion across generations. While historically integral to Caribbean identity, these practices are undergoing noticeable changes influenced by various sociopolitical and environmental factors, such as climate events, economic fluctuations, urbanisation, and changing family structures. Contemporary trends in food sharing practices indicate a move towards more convenience-oriented and processed foods, with reduced time spent cooking, and a decline in the use of food sources such as backyard gardens, fishing, and marketplaces, with a preference for supermarkets. These trends, particularly observed among younger generations, align with the ongoing nutrition transition in the Caribbean region [6, 7]. While a transition in these practices towards less healthy diets will increase the risk of many NCDs, the intrinsic cultural, social, and emotional benefits of food sharing could also positively impact health outcomes in the Caribbean region. Future research investigating the dynamic relationships between food sharing practices and wider social and food systems in the Caribbean region is needed.

### Limitations

Our sample did not achieve a balanced distribution of males and females (3 males and 17 females). Consequently, we did not conduct specific analyses based on sex or gender. Nevertheless, this limitation prompts reflection on why so many more women-identifying people volunteered for our study than men. This bias in the sample could be related to societal norms that often associate food sourcing and preparation with women, potentially making them more willing or comfortable to discuss food sharing practices. Another possible explanation is that women are generally more willing or able to participate in population-based health research than men, as observed in our previous work [24, 25], and in national health surveys in the region, such as the Barbados Health of the Nation Survey [49]. Both potential explanations would be interesting to explore and address in future research.

We recruited participants through a university’s online campus. E-learning offers significant benefits in terms of time, flexibility, and cost savings for students, such as reduced transportation and accommodation expenses, as well as an improved work-study-life balance. We believe that using this platform helped us reach a more diverse socioeconomic sample compared to on-site learning. However, we also acknowledge that targeting our recruitment through participants enrolled in online university programs—who face associated fees and require good internet connectivity—might have skewed our sample toward higher socioeconomic groups or, at least, upwardly mobile families. This might have influenced our findings; for example, participants could have been less likely to engage in food sharing practices, such as bartering, as a coping mechanism for severe food insecurity.

Our study was limited to a relatively small number of settings in the English-speaking Caribbean. While it is plausible that similar findings would be found in other English-speaking Caribbean settings, whether this is the case requires further research. Similarly, further research is needed to explore the relevance of our findings to other, non-English-speaking, parts of the Caribbean region. Future research exploring food sharing practices through a gender lens, as well as research including specific analyses based on setting (rural-urban), would be valuable.

## Electronic supplementary material

Below is the link to the electronic supplementary material.

Supplementary Material 1

Supplementary Material 2

Supplementary Material 3

## Data Availability

All data generated or analysed during this study are included in the body of this article or under additional files.
