# Supplementary material for: An exploratory case study of food sharing practices in Caribbean countries through a transition lens using intergenerational dyad interviews
Source: Global Health. 2024 Dec 24;20:88. doi: 10.1186/s12992-024-01094-0 (PMC11669230; doi:10.1186/s12992-024-01094-0)
Supplement: Supplementary file 1 — Supplementary Material 1 [file 12992_2024_1094_MOESM1_ESM.pdf]

Consolidated criteria for reporting qualitative studies (COREQ): 32-item checklist

**No. Item**	**Guide questions/description**	**Reported on Page #**
**Domain 1: Research team and reflexivity**		
Personal Characteristics		
1. Inter viewer/facilitator	Which author/s conducted the interview or focus group?	#9 (manuscript) and #6 (additional file 2)
2. Credentials	What were the researcher's credentials? E.g. PhD, MD	#1-2 (additional file 2)
3. Occupation	What was their occupation at the time of the study?	#1-2 (additional file 2)
4. Gender	Was the researcher male or female?	#1-2 (additional file 2)
5. Experience and training	What experience or training did the researcher have?	#7 (additional file 2)
Relationship with participants		
6. Relationship established	Was a relationship established prior to study commencement?	#7-9 (manuscript) and #8-11 (additional file 2)
7. Participant knowledge of the interviewer	What did the participants know about the researcher? e.g. personal goals, reasons for doing the research	#8-11,20-21,31-32 (additional file 2)
8. Interviewer characteristics	What characteristics were reported about the interviewer/facilitator? e.g. Bias, assumptions, reasons and interests in the research topic.	#7 (additional file 2)

**Domain 2: study design**		
Theoretical framework		
9. Methodological orientation and Theory	What methodological orientation was stated to underpin the study? e.g. grounded theory, discourse analysis, ethnography, phenomenology, content analysis	#9 (manuscript) and #13,42 (additional file 2)
Participant selection		
10. Sampling	How were participants selected? e.g. purposive, convenience, consecutive, snowball	#7-8 (manuscript) and #8-10 (additional file 2)
11. Method of approach	How were participants approached? e.g. face-to-face, telephone, mail, email	#9 (manuscript) and #8-11 (additional file 2)
12. Sample size	How many participants were in the study?	#8 (manuscript) and #41 (additional file 2)
13. Non-participation	How many people refused to participate or dropped out? Reasons?	#41 (additional file 2)
Setting		
14. Setting of data collection	Where was the data collected? e.g. home, clinic, workplace	#9 (manuscript) and #41-42 (additional file 2)
15. Presence of non-participants	Was anyone else present besides the participants and researchers?	#41-42 (additional file 2)
16. Description of sample	What are the important characteristics of the sample? e.g. demographic data, date	#8 (manuscript) and #7-8 (additional file 2)
Data collection		
17. Interview guide	Were questions, prompts, guides provided by the authors? Was it pilot tested?	#17-24 (additional file 2)
18. Repeat interviews	Were repeat interviews carried out? If yes, how many?	N/A
19. Audio/visual recording	Did the research use audio or visual recording to collect the data?	#9 (manuscript) and #3,10,12,42 (additional file 2)
20. Field notes	Were field notes made during and/or after the interview or focus group?	#9 (manuscript) and #42 (additional file 2)
21. Duration	What was the duration of the interviews or focus group?	#9 (manuscript) and #41 (additional file 2)
22. Data saturation	Was data saturation discussed?	N/A
23. Transcripts returned	Were transcripts returned to participants for comment and/or correction?	#42 (additional file 2)
**Domain 3: analysis and findings**		
Data analysis		
24. Number of data coders	How many data coders coded the data?	#9 (manuscript)
25. Description of the coding tree	Did authors provide a description of the coding tree?	#1 (additional file 3)
26. Derivation of themes	Were themes identified in advance or derived from the data?	#9 (manuscript) and #4-8 (additional file 3)

27. Software	What software, if applicable, was used to manage the data?	#9 (manuscript)
28. Participant checking	Did participants provide feedback on the findings?	#42 (additional file 2)
Reporting		
29. Quotations presented	Were participant quotations presented to illustrate the themes/findings? Was each quotation identified? e.g. participant number	#10-27 (manuscript)
30. Data and findings consistent	Was there consistency between the data presented and the findings?	#10-27 (manuscript) and #1-8 (additional file 3)
31. Clarity of major themes	Were major themes clearly presented in the findings?	#10,15,19,22 (manuscript) and #6-8 (additional file 3)
32. Clarity of minor themes	Is there a description of diverse cases or discussion of minor themes?	#14,15,18-20,22,25 (manuscript)
