# Supplementary material for: An exploratory case study of food sharing practices in Caribbean countries through a transition lens using intergenerational dyad interviews
Source: Global Health. 2024 Dec 24;20:88. doi: 10.1186/s12992-024-01094-0 (PMC11669230; doi:10.1186/s12992-024-01094-0)
Supplement: Supplementary file 2 — Supplementary Material 2 [file 12992_2024_1094_MOESM2_ESM.pdf]

Study Protocol

Exploring food sharing practices in Caribbean Small Island Developing States: a qualitative study

Investigators and institutional affiliations
Anna Brugulat-Panés, MRC Epidemiology Unit
Louise Foley, MRC Epidemiology Unit
Cornelia Guell, University of Exeter
Nigel Unwin, MRC Epidemiology Unit
Madhuvanti (Maddy) Murphy

Funder
Economic and Social Research Council Cambridge Doctoral Training Partnership
(Grant reference ES/J500033/1)

Local Ethical approval

The University of West Indies
Open Campus Research Ethics Committee
(Ref. CREC-OC.0110/07/2022)

Sponsor Ethical approval

Cambridge Psychology Research Ethics Committee
(PRE.2022.087)

Table of Contents

1. General Information 1
2. Lay Summary 3
3. Background and Rationale 3
4. Justification 4
5. General and Specific Objectives 5
6. Study Design and Methodology 6
   Overall design 6
   Study setting 6
   Data collection method 7
   Researcher training and experience 7
   Study population 7
   Sample size determination 8
7. Sampling Procedure 8
   Recruitment 9
   Sample identification 9
8. Data Collection Procedure 10
9. Confidentiality 11
   Participant withdrawal criteria/Consent 11
   Data storage 11
   Data management 12
   Ethical considerations 13
10. Funding 13
   Time duration of the project 13
   Budget summary 13
   Justification of the budget 14
11. Expected Outcomes and Dissemination 14
   Publications 14
   Other dissemination materials 14
   Engagement 14
12. References 14
13. Appendices 17
   A. Topic Guide 17
   B. List of eligible Caribbean SIDS 26
   C. Study Advert 27
   D. Registration of Interest Electronic Form 28
   E. Participant Information Sheet (PIS) 32
   F. Consent and Signature 38
   G. Transcription Specification Sheet 39
   H. Letter of local ethics approval 40

I. Letter of Cambridge ethics approval 41
J. Extended description of Methods. 42

1. General Information

Protocol title	Exploring food sharing practices in Caribbean Small Island Developing States: a qualitative study
Protocol number	Version 0.2
Date	Setembre 2022
Sponsor	University of Cambridge
Authorised signatory of protocol	Dr. Louise Foley
Sponsor's medical expert	N/A
Investigative team	**Investigators**<br>Anna Brugulat-Panés (ABP)<br>PhD Student<br>MRC Epidemiology Unit<br>University of Cambridge<br>anna.brugulat@mrc-epid.cam.ac.uk<br><br>Dr. Louise Foley (LF)<br>Senior Research Associate *(PhD supervisor)*<br>MRC Epidemiology Unit<br>University of Cambridge<br>louise.foley@mrc-epid.cam.ac.uk<br><br>Dr. Cornelia Guell (CG)<br>Senior Lecturer and Centre Co-Director *(PhD supervisor)*<br>European Centre for Environment and Human Health<br>University of Exeter<br>C.Guell@exeter.ac.uk<br><br>Prof. Nigel Unwin (NU)<br>Professor in Public Health and Epidemiology *(PhD advisor)*<br>MRC Epidemiology Unit<br>University of Cambridge<br>nigel.unwin@mrc-epid.cam.ac.uk<br><br>Dr. Madhuvanti (Maddy) Murphy (MM)<br>Senior Lecturer *(PhD advisor)*<br>The George Alleyne Chronic Disease Research Centre<br>University of West Indies<br>madhuvanti.murphy@cavehill.uwi.edu
Trial sites	MRC Epidemiology Unit<br>University of Cambridge<br>Institute of Metabolic Science<br>Box 285<br>Cambridge Biomedical Campus

	Hills Road<br>Cambridge<br>CB2 0QQ<br>United Kingdom<br>Ph: +44 01223 330315<br><br>The European Centre for Environment & Human Health<br>University of Exeter<br>Medical School<br>Knowledge Spa, Royal Cornwall Hospital<br>Truro<br>TR1 3HD<br>United Kingdom<br>Ph: +44 01872 258138<br><br>The George Alleyne Chronic Disease Research Centre<br>Caribbean Institute for Health Research<br>"Avalon", Jemmott's Lane<br>Bridgetown<br>St.Michael<br>BB11115<br>Barbados, West Indies<br>Ph: (246) 426-6416
Qualified physician	N/A
Clinical laboratory	N/A
Operational team	Anna Brugulat-Panés
Study steering committee	This study forms part of the Global Diet and Activity Research Group and Network (GDAR). The GDAR network steering group will act as a study steering committee, providing general oversight and advice on scientific and operational issues
Contributions of protocol authors	Develop protocol<br>1. All investigators<br>2. With assistance from Annie Schiff (Global Health Project Coordinator, MRC Epidemiology Unit) and Rebecca Margieson (Qualitative Research Study Coordinator, MRC Epidemiology Unit)
Role of the funder	The funders have provided financial support for the development and implementation of the study. The funder will have no input into the development of this protocol or the interpretation or publication of results

2. Lay Summary

This study aims to explore the role of generational, life course and socio-cultural factors in contemporary food sharing practices and experiences in the Caribbean. This study has been informed by a systematic scoping review of peer-reviewed and grey literature on types of food sources for consumption in this context.

Virtual interviews will be conducted with ten to fifteen intergenerational dyads (20-30 people) involved in food sharing practices in the Caribbean Small Island Developing States. The target group will be students enrolled in online programmes at the University of West Indies Open Campus and a family-relative that will form the intergenerational dyad.

Data collection will occur online using the free videoconference platform Teams or Zoom, and data analysis will occur at the MRC Epidemiology Unit, University of Cambridge in the United Kingdom.

Any spreadsheets and documents containing contact details of the participant, consent forms and recordings will be kept separate from each other to ensure data confidentiality. Study documents, audio files of interviews and their transcripts will be held at the MRC Epidemiology Unit following their data management and protection procedures, as explained in detail later in this application, to satisfy the requirements of the General Data Protection Regulations. Given that qualitative data cannot be easily anonymised, data protection will be carefully considered, and data uploaded to the Secure Research Drive as soon as possible after the interviews. After checking for quality and completeness of the uploaded recordings, any other versions will be deleted.

A broad project timeframe is as follows:

- Develop study protocol: Jan 2022- Mar 2022
- Develop study documents: Feb 2022 – May 2022
- Submit local ethics approval (i.e., UWI): Jun 2022 – Aug 2022
- Submit sponsor ethics approval (i.e., Cambridge University): Sep 2022
- Other legal requirements including Cambridge sponsorship and insurance: Sep 2022
- Participants' recruitment and data collection: Sep 2022 – Dec 2022
- Data analysis and synthesis: Sep 2022 – Jan 2023
- Dissemination: Jan 2023 – Feb 2023

3. Background and Rationale

Populations in Caribbean countries experience high rates of noncommunicable diseases (NCDs), especially obesity and diet-related chronic diseases, with some of the highest prevalence rates in the world (1,2). These populations also suffer from NCDs' associated premature (before the age of 70 years) mortality, for example in the Bahamas, in 2016 one in seven adults died prematurely from NCDs (24). Most of the Caribbean countries are highly dependent on external and costly markets thus facing food insecurity and a rapid nutritional transition (2-4). Inadequate availability and access to food have further impacts on diets which are increasingly dominated by cheap

processed imported foods (5). Some of the major factors driving these challenges are geophysical and structural constraints, climate change, profound changes in food systems associated with globalisation and market liberalisation agreements, and inadequate local agriculture systems shaped by legacies of colonialism (6-8, 27,28).

There is some, albeit limited, existing research on food sources and supply in the Small Island Developing States (SIDS) context (9,10,26) that is already being used in this study. In addition, this study has been informed by an ongoing systematic scoping review on types of food sources for consumption in the SIDS context. A recurring theme is the existence of different food sharing practices as a food source with a potential role in dietary diversity and food security, as seen in urban and rural communities in Saint Vincent and the Grenadines (9,10). Reviewed evidence indicates that; there might exist a great heterogeneity of food sharing practices involving consumption of local foods (11-16) which could have a role in reducing dependency on food imports in the Caribbean and in turn contributing to long term NCD prevention (29); foods shared through these practices are grown and reared in family farms, backyards, or community gardens (17-23) that use traditional ecological knowledge and diversified farming practices; there may be potential links between food sharing practices and the consumption of more sustainable and diverse diets that maintain cultural knowledge on the way to food sovereignty, to healthier diets and to addressing the growing burden of NCDs in the region. Moreover, food sharing practices seem to capture the importance of people’s social interactions and networks in accessing foods and ensuring food security via non-monetary means. For example, evidence from Cuba showed how through informal social networks the local population could access certain food products (lobster) which by law were only intended for tourism and export (17).

This deserves further attention as it could be used to increase Caribbean countries' resilience to natural disasters and economic future shocks, as recently seen during the COVID-19 pandemic when food sharing practices within family and the community were used in the Caribbean region as a coping strategy to ensure food security and access to livelihoods (16). However, there seems to be a significant gap in evidence and understanding in these informal food practices and how they may be changing over generations and life course. Evidence suggests this is an important part of understanding local diets and therefore in the next section we propose the following aim and objectives for this study.

4. Justification

This qualitative study forms part of a broader mixed-method PhD project. The overarching aim of the PhD project is to develop understanding of food sovereignty in SIDS with a focus on the role of the informal economy and food sharing practices. I will achieve this through the following preliminary research questions:

1. What are the existing sources of food for consumption in SIDS in terms of their variety, extent, and nature? (Study 1)
2. What is the role of generational, life course and socio-cultural factors in contemporary food sharing practices and experiences in Caribbean SIDS? (Study 2)

3. What are the trends in supply and availability of different food types in SIDS, including whether they are produced locally or imported, and their ecological associations with NCD risk? (Study 3)
4. How do food sharing practices identified in Study 1 and Study 2 relate to the wider foodscape and food system in SIDS and in particular to NCDs? (Study 4)

I plan to address these preliminary research questions through the following proposed research studies:

1. Evidence synthesis consisting of mixed-method systematic scoping review (Study 1)
2. Qualitative study consisting of primary data analysis of interviews (Study 2)
3. Quantitative study consisting of ecological analysis of routinely available data (Study 3)
4. Participatory systems mapping study (Study 4)
5. Synthesis of all emerging findings following principles of process tracing (Discussion Chapter)

This qualitative study will constitute Study 2 of the PhD project and it will contribute to the PhD research questions 1, 2 and 4.

5. General and Specific Objectives

The objectives for this qualitative study will therefore be:

General objective

To explore the role of generational, life course and socio-cultural factors in contemporary food sharing practices and experiences in Caribbean Small Island Developing States

Specific objectives

The general objective will be achieved through the following specific objectives:

1. To explore food sharing practices and experiences in intergenerational dyads (family-related adults from different generations):
   a) to get a better understanding of the fundamentals of these practices
   b) to investigate their potential to improve diet and food security as well as other economic, social, and environmental benefits
2. To explore the meaning and cultural value people place on food sharing practices and how these may differ between generations
3. To understand what influences people’s food sharing practices, including global and life events, and how these may differ between generations
4. To investigate local perspectives on the potential wider role of food sharing practices in the context of climate change resilience, sustainability, and food agency.

6. Study Design and Methodology

Overall design

A small qualitative study consisting in online interviews with ten to fifteen intergenerational dyads - formed by family-related adults - involved in food sharing practices in the Caribbean SIDS.

For the entire duration (Jan 2022 – Feb 2023), this study will take place at the MRC Epidemiology Unit, University of Cambridge (United Kingdom) and will involve online data collection from adult participants living in different Caribbean SIDS. For that, the interviews will take place virtually using the free videoconferencing platform Teams or Zoom. We plan to access participants through the University of West Indies (UWI) Open Campus University for its reach across different Caribbean islands and assuming adequate Internet access of participating students in this online study. Data collection, storage and analysis will take place at the MRC Epidemiology Unit as the investigators from the research study team doing these activities will be based there.

Study setting

We will seek recruitment of participants from several Caribbean countries through the UWI Open Campus. This is the virtual campus of the UWI which also offers onsite facilities in seventeen English-speaking Caribbean SIDS. These are: Anguilla, Antigua & Barbuda, Bahamas, Barbados, Belize, Bermuda, British Virgin Islands, Cayman Islands, Dominica, Grenada, Jamaica, Montserrat, St. Kitts & Nevis, St. Lucia, St. Vincent & the Grenadines, Trinidad & Tobago, Turks & Caicos.
The UWI Open Campus University:

- welcomes applications from residents in these Caribbean SIDS as well as international applicants.
- offers undergraduate and graduate programmes which are fully taught in English. Applicants whose native language is not English are required to provide a minimum TOEFL examination score of 500 or its IELTS equivalent.
- has fees which can be paid in the local currency or in Unites States Dollars and that have an average cost of US$660.00 per one-year course. Scholarships and financial aid are offered.

Given the characteristics of the UWI Open Campus explained above, we aim:

- to reach participants living in different Caribbean SIDS, including difficult-to-reach settings. By this, we mean for example participants living in geographical areas such as remote islands which would be very costly to visit in-person by the investigators.
- to reach participants from diverse socio-economic backgrounds, ages, and gender. E-learning can have significant time and flexibility benefits as well as economic benefits for students, such as less costs in transport or accommodation, which we believe it will contribute towards reaching a more diverse sample.
- to reduce the burden of potential barriers to conduct this study such as language barriers, travel restrictions and research-associated costs.

Data collection method

We aim to use dyadic interviews as the method to collect the study data. Dyadic qualitative interviewing brings two people at the same time to respond together to open-ended research questions. Dyadic interviews are relationship-based interviews where participants interact with each other in a conversation guided by a semi-structured interview (21). The method allows each participant to develop personal narratives around a research topic while it stimulates new ideas that might otherwise be missed (22).

An investigator of this study based in Cambridge (ABP) will conduct the interviews online using the video teleconferencing software Zoom or Teams. Each dyadic interview will be planned to last for about 60 minutes and will follow the topic guide included in Appendix A. During the interviews, we will explore how participant's experience food sharing practices, with attention to how these practices may have changed between inter-family generations, and their reflections on the role of food sharing practices on issues such as food sovereignty and climate change resilience.

Interviews will be recorded by ABP using the appropriate recording equipment (i.e., Dictaphones) available at the MRC Epidemiology Unit. We will use two recorders in case one recorder fails. These will be collected and tested well in advance of planned interviews.

Researcher training and experience

Before joining the Unit, ABP received formal training in qualitative research methods during her MSc in Global Public Health Nutrition. Putting her knowledge into practice, she conducted her master's project and dissertation titled 'The Role of Agroecology on Food Nutrition and Security in Cuba: a Mixed-Methods Approach'. Leading this project, ABP undertook field data collection in Cuba over a three-week period. Employing a mixed-methods approach, the project incorporated literature review techniques and qualitative research methods. The qualitative segment involved conducting semi-structured interviews with six key informants from public health, agriculture, and trade and economy sectors, as well as structured interviews with the community using Household Dietary Diversity Score (HDDS) and Household Food Insecurity Access Scale (HFIAS) questionnaires within a small purposive sample of 14 participants. This project provided ABP with experience in navigating the ethics review process for overseas work, including considerations for local and sponsor ethics, funding, data management, translation, and transcription. Subsequently, ABP participated in an online focus groups workshop at GDAR led by Dr. Ishtar Govia from the University of West Indies to refresh her previous training. Continuing her formal training in qualitative research methods, ABP furthered her education through the University of Cambridge and the ESRC-DTP training program. She completed an online course in Ethnographic Methods and joined the Qualitative Research Group at the Unit. Additionally, since last January, ABP has attended the online Qualitative Research Workshop at the University of Exeter delivered by Dr. Cornelia Guell to enhance her proficiency in qualitative methods.

Study population

We aim for a diverse purposeful sample that includes adults from different socio-economic backgrounds, gender, age and living areas.

Both individuals involved in the dyadic interview must:

- be Caribbean SIDS's residents. A list of Caribbean SIDS is provided in Appendix B.
- be adults (i.e.,18 years or older)
- be able to communicate in English, or Spanish[1]
- be or had been involved in food sharing practices in one way or another.

  Any of the following will be included:
  - Food producers: small-scale/family/subsistence farmers or home gardeners who share their food surplus (i.e., food givers)
  - Individuals who receive food from others in exchange of labour, or of other foods, or as gifts. (i.e., food receivers)
  - Individuals volunteering in organisations involved in food sharing activities such as social agricultural projects, food rescue, food redistribution, home-cooking, and meal sharing.
- have an intergenerational relationship (i.e., parental, or other familial)

  Examples of these are:
  - UWI Student or alumni + elder family-relative such as parent or grandparent
  - UWI Student or alumni + younger adult family-relative such as child or grandchild

At least one person of the pair needs to:

- have access to internet and the adequate infrastructure to participate in online interviews

Sample size determination

We aim for a sample size of about ten to fifteen dyadic interviews (i.e., about twenty to thirty individuals). Small interview numbers are justified in exploratory qualitative studies like this one if they provide sufficient rich understanding while allowing for an in-depth inquiry of the topic explored.

7. Sampling Procedure

It has been agreed by the UWI Open Campus Research Ethics Committee to follow the following steps for recruitment:

Step 1. Dissemination of study advert with link to electronic form
Step 2. Electronic form to register interest

[1] The researcher conducting data collection is a native Spanish speaker and could provide documents in Spanish and conduct interviews in Spanish on request if that was the preferred language by the participants.

Step 3. Participant information sheet (PIS) and informed consent sent by email to only the interested people using REDCap system. Some people may just go ahead and sign the e-consent at this step.
Step 4. Contact by email or videoconference call to answer any questions and confirm eligibility
Step 5. Sign e-consent form using REDCap system
Step 6. Schedule date and time for virtual interview

Recruitment

We have developed a study advert (Appendix C) which will be disseminated widely to seek expressions of interest. The dissemination will be done through the UWI Open Campus internal communication channels such as an ‘all-students’ type of email, the website, social media pages and the Campus News. In addition to these channels, we will our research partners at UWI to share the study advert widely among their networks at the University (Step 1). In doing this, we will make sure that potential participants will be undergraduate or graduate students, or alumni of the UWI Open Campus University and that, given the characteristics of this Campus, potential participants will be likely to speak English and have the equipment needed to participate in online interviews.

Sample identification

The study advert will provide a link to a short electronic form that will register people’s interest in participating. This electronic form (Appendix D) will ask for basic socio-demographic information such as age, island of residence, and ability to speak English or Spanish, to provide an initial screening of participant’s eligibility, and will ask for a contact email. The electronic form will be developed with the assistance of the Data Management Team at the MRC Epidemiology Unit and will use the REDCap system (Step 2).

Contact with interested individuals will follow via email. We will get in touch with interested individuals only to provide them with the PIS (Appendix E) and the informed consent form (Appendix F). These documents will explain the purpose of the study, what participants will be required to do, and how they will be involved (Step 3). Some people may just go-ahead at this step and sign the e-consent form sent via REDCap system.

Further contact with interested individuals will follow via email or videoconference calls (according to potential participants’ preference) to answer any remaining questions, to ensure the eligibility criteria is met, and to help to identify the inter-generational member, if needed (Step 4).

Interested individuals and their intergenerational relatives who confirm their willingness to participate will be asked to sign an e-consent form as a confirmation of consent to be interviewed. Both members of the dyad must agree to participate and provide consent. The electronic consent form will be developed with the assistance of the Data Management Team at the MRC Epidemiology Unit and will use the REDCap system (Step 5). This will consist in a statement of

confirmation that participants will need to sign online, and they will not need to download or print or scan.

After consent, we will agree with the selected participants on a date and time of their convenience to arrange the virtual interview (Step 6). Prior to starting the virtual interviews, all participants will be reminded of their right to withdraw from the study at any time and another opportunity to ask questions will be given.

8. Data Collection Procedure

Before attending the online interview, we will ensure that all participants have received a PIS and informed consent or will provide one if they have not. We will check that all participants have read and understood the information about the study and explain the format of the data collection and how long it is expected to last. We will make it clear that the dyadic interview will be recorded, but that participants can stop the interview at any time and can request that the recording be deleted. Prior to the interviews, all participants will be asked to complete the online consent form (right before the interview or in a separate previous meeting); all participants will be able to ask questions and be reminded that they can withdraw at any time. Signed e-consent forms and any other documents containing personal identifiable data will be uploaded to the Secured Research Drive (SRD) at the MRC Epidemiology Unit. This is a designated 'safe haven' space that has been assessed against the NHS Digital Data Security and Protection Toolkit and has achieved 'Standards Met.

Prior to starting the virtual interview, we will make sure that we have all equipment and e-documents ready to be used, including the interview topic guide, and recording equipment for audio capture. Interviews will be recorded using the appropriate recording equipment (i.e., Dictaphones) available at the MRC Epidemiology Unit. Please note, the MRC Epidemiology Unit's procedures for data protection do not allow for the Teams or Zoom recording function to be used. We will use two recorders in case one recorder fails or is of poor quality. These will be collected and tested well in advance of planned interviews.

When the session begins, we will remind the volunteers that the interview is being recorded and will start the audio recorder(s). We will state the study code, participants ID, date and time at the start of the recording.

During the interview, we will follow the study-specific protocol interview topic guide. The interview will be conducted online using the free videoconferencing software Teams or Zoom according to the volunteer's preference. Each dyadic interview will be planned to last for about 60 minutes. During the interviews, we will explore how participants experience food sharing practices, with attention to how these practices may have changed between inter-family generations, and their reflections on the role of food sharing practices on issues such as food sovereignty and climate change resilience.

At the end of the interview, the recording will be downloaded straight to the SRD, then it will be sent to the transcription company, then once returned, data management will store it back on the SRD. The uploaded recording will be checked for quality and completeness, and the recorder held version will then be deleted. Anonymised transcripts will be then placed by the data management team in a limited access folder on the group drive.

9. Confidentiality

Participant withdrawal criteria/Consent

The UWI Open Research Ethics Committee requested that we use their consent forms and wording as per Appendix F.

E-consent will be collected from all participants using the REDCap system following the process below:

The study team will send via email a copy of the PIS and informed consent form to individuals who register their interest in participating in the study. Further conversations will follow up either via email or virtual call according to interested people’s preference. The process will take the form of a discussion between the study team and the interested potential participants. Ample time will be allocated to discuss the study in detail and address any questions the potential participants may have. The time required to do this procedure effectively will vary for each participant, however the discussion will be long enough to ensure all topics are covered and understood by the participant.

We will explain to the volunteers how long their interview will last, what will happen to them during their interview, what happens following their interview and what is done with their data. We will allow ample opportunity for the volunteer to ask questions and address any concerns they may have. We will make it clear to the volunteers that their participation is voluntary and they are free to withdraw at any time. We will also allow time for the volunteer to read the consent form. We will ask the potential participant to read through the consent form and initial the boxes that they are happy to consent to.

When the potential participants have understood the topics above, we will ask the potential participant if they would like to participate in the study. If the potential participant is keen to enter the study, we will provide them with the link to the e-consent form. Both members of the dyad must agree to participate and provide consent.

We will ensure all necessary boxes on the right-hand side of the e-consent form are initialled by the volunteer, and that potential participants have signed and dated the e-consent form. Once the potential participant has signed and dated the form we will sign and date the form immediately.

Data storage

We will only collect electronic data. E-consent forms will be stored for archival purposes, which will be kept separated from participant study data at all times. All electronic forms will be prepared using the REDCap system - a secure web application for building and managing online surveys and databases.

All qualitative interviews will be conducted online in a private space such as the participant's home. After the interviews, audio files of interviews will be held at the MRC Epidemiology Unit following their data management and protection procedures. Any spreadsheets and documents containing contact details of the participant, consent forms and recordings will be kept separate from each other to ensure data confidentiality.

Given that qualitative data cannot easily be anonymised, the audio files and their transcripts as word documents will be uploaded to the SRD as soon as possible after the interviews. After checking for quality and completeness of the uploaded recordings, any other versions will be deleted.

At the end of the study, the confidential records will be stored securely at the MRC Epidemiology Unit for 20 years in accordance with the University of Cambridge data policies and then destroyed.

Data management

Data transfer for this study will not be necessary as the analysis will take place at the MRC Epidemiology Unit by the same investigators that will collect, record and held the data. However, we have made clear in the consent forms that there may exist the possibility of sharing the data with UWI partners for other research purposes. This has been mentioned in the PIS and consent forms following the guidance received from the UWI Open Research Ethics Committee.

We do not expect any data translation to be required as per the participant eligibility criteria. The meaning of any local words used by the participants will be clarified during the interviews. However, if there were any excerpts that we could not understand, we would ask for assistance from our research partners at UWI.

A transcription company frequently used by the MRC Epidemiology Unit called 'The Typing Works' will transcribe all interviews verbatim[2]. This is a trusted company with experience of transcribing Caribbean-English accents. For that, we will prepare a transcription specification sheet containing data specific details (Appendix G). The data management team at the Unit will arrange the transfer of individual files and the specifications to the company via a secure file transfer protocol. Transcripts as word documents will be return to the data management team following the same

[2] Any potential interviews held in Spanish will be transcribed by the researcher conducting data collection who is a native Spanish speaker

protocol who will upload them into the SRD with limited access to the investigators of this study for their analysis. Once received, the investigators will listen to the recordings and check for any errors in the transcripts. We will anonymise data from the transcripts by removing all personal information that could directly identify an individual to protect participant confidentiality. Pseudonyms or generic descriptors will be used to edit identifying information, rather than blanking-out information. Any identifying information that is not relevant to the analysis will be removed. In the case of needing to share the transcripts with the UWI partners for other research purposes, this will be arranged by the data management team and sent as an encrypted file via a secure file transfer protocol.

The transcripts from the interviews will be analysed using the NVivo Qualitative Data Analysis Software and following an interpretative thematic analysis method which will follow five main steps: familiarisation with the data, theme and subtheme identification, coding assignation, chart representation and interpretation (23). Several members in the study team will do this, and we will discuss progress with the collaborators in UWI.

Ethical considerations

Local ethical approval for this study has been obtained from the Open Campus Research Ethics Committee at the University West Indies (Appendix H). Sponsor ethical approval will be sought from the University Ethics board of Psychology Research Ethics Committee (PREC).

10. Funding

Time duration of the project

The estimated total timeframe for this study is fourteen months (Jan 2022 - Feb 2023). This timeframe includes the time needed to develop the study documents, to get local and sponsor ethics approvals and other legal requirements, to conduct recruitment of participants and data collection, and to complete data analysis and synthesis.

A broad project timeframe is as follow:

- Develop study protocol: Jan 2022- Mar 2022
- Develop study documents: Feb 2022 – May 2022
- Submit local ethics approval (i.e., UWI): Jun 2022 – Aug 2022
- Submit sponsor ethics approval (i.e., Cambridge University): Sep 2022
- Other legal requirements including Cambridge sponsorship and insurance: Sep 2022
- Participants' recruitment and data collection: Sep 2022 – Dec 2022
- Data analysis and synthesis: Sep 2022 – Jan 2023
- Dissemination: Jan 2023 – Feb 2023

Budget summary

This study will not require any travel from the investigators and/or participants as data collection will consist of online interviews through free platforms such as Zoom or Teams. We do not anticipate any costs related to technological infrastructure such as laptops, internet access, mic-camera sets, etc. as investigators and eligible participants for the study will already have the adequate setup for online teaching. Participants in this study will be part of the UWI Open Campus, which is entirely taught in English, thus we do not expect any translation-associated costs. Specific training needs or refreshment on qualitative research that may be required by the investigator/s carrying data collection and analysis will be covered by the University of Cambridge and the ESRC DTP free training programmes.

We anticipate having costs associated with data transcription and other resources to support the research which have been specified in the next section.

Justification of the budget

Budget to cover the following costs will be sought trough the MRC Epidemiology Unit and through the Economic Social Research Council Doctoral Training Partnership.

- Transcription of qualitative interviews: between c.£850 and £1275 plus VAT, depending on the final word count. [this is based on 10-15 interviews of a duration length of about 60 minutes each]
- NVivo software licence fee and voice recorders: £0 [already available at the MRC Epidemiology Unit]
- Data Management (Grade 7; spine point 7) at 100% FTE x 2 days over the lifetime of the research project to support this study in setting up secure consent forms via REDCap and managing and storing the data. This equates to £268.

11. Expected Outcomes and Dissemination

Publications

The University of Cambridge coordinates this study consisting in research study members from other participating universities. Any wholly reliable interim findings will be disseminated rapidly to stakeholders and participants. There will be group and single authorship recognising the contribution of all national and local investigators. Data analysed from this study will be shared at conferences and related workshops.

Other dissemination materials

Materials such as policy briefs, infographic or factsheets could be developed for further dissemination and public engagement with experts at CARICOM, PAHO and other regional UN agencies and NGOs working in Caribbean small islands. The supervisory team has a particularly close relationship to the Healthy Caribbean Coalition, a CARICOM-wide alliance of health NGOs.

Engagement

This study is important work that could contribute to ongoing global discussions on issues such as environmental sustainability, resilience to climate change, food and nutrition security, inclusive systems, and community empowerment. There is opportunity for sharing relevant findings across network groups and advocates in the Caribbean region and the UWI community. Involvement from relevant stakeholders could also be considered in next steps, for example through their participation in a group model building workshop, to relate the findings from this application to the wider foodscape and food system, and in particular to NCDs. There is also potential to create links for further collaborative work with UWI students in public health or relevant disciplines to expand and follow up with findings from this application.

12. References

1. Abubakar, I. I., Tillmann, T., & Banerjee, A. (2015). Global, regional, and national age-sex specific all-cause and cause- specific mortality for 240 causes of death, 1990-2013: a systematic analysis for the Global Burden of Disease Study 2013. Lancet, 385 (9963), 117-171.
2. Global Nutrition Report. (2020). Available online: https://globalnutritionreport.org/reports/2020-global-nutrition-report/ (accessed on 9 November 2021).
3. Popkin, B. M., Adair, L. S., & Ng, S. W. (2012). Global nutrition transition and the pandemic of obesity in developing countries. Nutrition reviews, 70(1), 3-21.
4. Food and Agriculture Organization of the United Nations. (2014). Food Security and Nutrition in Small Island Developing States. FAO: Rome, Italy.

5. Swinburn, B. A., Sacks, G., Hall, K. D., McPherson, K., Finegood, D. T., Moodie, M. L., & Gortmaker, S. L. (2011). The global obesity pandemic: shaped by global drivers and local environments. The Lancet, 378(9793), 804-814.
6. United Nations. Department of Economic and Social Affairs. Sustainable Development. Small Island Developing States. Available online: https://sdgs.un.org/topics/small-island-developing-states (accessed on 9 November 2021).
7. Baldacchino, G. (2014). Small island states: Vulnerable, resilient, doggedly perseverant or cleverly opportunistic?. Études caribéennes, (27-28).
8. Roessler, P., Pengl, Y. I., Marty, R., Titlow, K. S., & van de Walle, N. (2020). The cash crop revolution, colonialism and legacies of spatial inequality: Evidence from Africa (No. 2020-12). Centre for the Study of African Economies, University of Oxford.
9. Haynes, E., Bhagtani, D., Iese, V., Brown, C. R., Fesaitu, J., Hambleton, I., ... & Unwin, N. (2020). Food sources and dietary
quality in small island developing states: development of methods and policy relevant novel survey data from the Pacific and
Caribbean. Nutrients, 12(11), 3350.
10. Guell, C., Brown, C. R., Iese, V., Navunicagi, O., Wairiu, M., & Unwin, N. (2021). "We used to get food from the garden."
Understanding changing practices of local food production and consumption in small island states. Social Science &
Medicine, 284, 114214.
11. FAO. 2012. Report of the FAO/SPC Pacific Islands Regional Consultation on the Development of Guidelines for Securing Sustainable Small-Scale Fisheries, Noumea, New Caledonia, 12–14 June 2012. FAO Fisheries and Aquaculture Report No. 1022. Rome. 32 pp. Available online: https://www.fao.org/3/i3063e/i3063e.pdf (accessed on 13 November 2021).
12. FAO. 2018. Family Farming in the Pacific Islands Countries. Available online: https://www.fao.org/documents/card/en/c/CA0305EN/ (accessed on 13 November 2021).
13. Gillett, R., & Tauati, M. I. (2018). Fisheries of the Pacific Islands: regional and national information. FAO Fisheries and Aquaculture Technical Paper, (625), I-400.
14. FAO. (2015). Growing Greener Cities. Urban and Peri-urban Agriculture in Latin America and the Caribbean. Antigua and Barbuda. Available online: https://www.fao.org/ag/agp/greenercities/en/GGCLAC/antigua_and_barbuda.html (accessed on 13 November 2021).
15. ShareCity. (2017). Thank you food sharers of Singapore. Available online: https://sharecity.ie/thank-food-sharers-singapore/ (accessed on 13 November 2021).
16. WFP (2021). Caribbean COVID-19 Food Security and Livelihoods Impact Survey - Round 3 - February 2021. Available online: https://docs.wfp.org/api/documents/WFP-0000125496/download/?_ga=2.262092557.1672205072.1637599530- 587590887.1637599530 (accessed on 13 November 2021).
17. ShareCity. (2019). Summer Edition: Food in Cuba!. Available online: https://sharecity.ie/food-in-cuba/ (accessed on 13
November 2021).
18. Darmadi, G. (2020). Fijians turn to bartering system as coronavirus shutdowns cause mass unemployment. Abc News. Available online: https://www.abc.net.au/news/2020-06-01/as-

unemployment-soars-fijians-turn-to-bartering-to-get-by/12303252 (accessed on 13 November 2021).
19. Schiefenhövel, W. (2014). On the human ethology of food sharing. AnthropologicAl review, 77(3).
20. Takasaki, Y. (2011). Targeting cyclone relief within the village: kinship, sharing, and capture. Economic Development and Cultural Change, 59(2), 387-416.
21. Morgan, D.L. et al. (2013) 'Introducing Dyadic Interviews as a Method for Collecting Qualitative Data', Qualitative Health Research, 23(9), pp. 1276–1284. doi:10.1177/1049732313501889.
22. Sexton Topper, P. and Bauermeister, J.A. (2021) 'Relationship Timelines, Dyadic Interviews, and Visual Representations: Implementation of an Adapted Visual Qualitative Technique', International Journal of Qualitative Methods, 20, p. 16094069211016708. doi:10.1177/16094069211016708.
23. Graneheim, U.H. and Lundman, B. (2003). Qualitative content analysis in nursing research: concepts, procedures and
measures to achieve trustworthiness. Elsevier. Nurse Education Today, 24, 105-112. Available from https://doi.org/10.1016/j.nedt.2003.10.001
24. World Health Organization. Noncommunicable Diseases Country Profiles; World Health Organization: Geneva, Switzerland, 2018.
25. Hickey, G. M., & Unwin, N. (2020). Addressing the triple burden of malnutrition in the time of COVID-19 and climate change in Small Island Developing States: what role for improved local food production?. *Food Security*, *12*(4), 831-835.
26. Bhagtani, D., Augustus, E., Haynes, E., Iese, V., Brown, C. R., Fesaitu, J., ... & Unwin, N. (2022). Dietary Patterns, Food Insecurity, and Their Relationships with Food Sources and Social Determinants in Two Small Island Developing States. *Nutrients*, *14*(14), 2891.
27. FAO. *FAO's Work with Small Island Developing States: Transforming Food Systems, Sustaining Small Islands*; FAO: Rome, Italy, 2019; Available online: https://www.fao.org/3/ca5170en/CA5170EN.pdf (accessed on 09 Aug 2022).
28. Smith, M.D.; Kassa, W.; Winters, P. Assessing food insecurity in Latin America and the Caribbean using FAO's Food Insecurity Experience Scale. *Food Policy* **2017**, *71*, 48–61.
29. Trujillo, A. G. (2015). The hefty challenges of food sovereignty's adulthood–Synthesis paper. *Canadian Food Studies*, *2*(2), 183-19

13. Appendices

A. Topic Guide

Introduction of study *[add short summary]; house rules: e.g., keeping all contributions confidential, no need for names/real names.*

Introduction of interview procedure: *approx. 60 minutes [check with participants on time available when scheduling each interview]; semi-structured interview with general areas we would like to cover but meant to be a guide; clarifying that they can put emphasis on particular topics and add topics we haven't covered; explaining that the interview is being tape-recorded and it will be transcribed– but no identifiable information kept.*

Individual consent - consent procedure:
Did you read and understand the information sheet and consent form? Can I explain anything? Do you have any questions? I would like to stress that we would not identify the specific source of comments in any write ups. *(Review participant information sheet and confirm e-sign consent form)*

Ice breaker - Introductions:
What is your relationship? Can you briefly introduce the other person?

**Main Questions**	**Possible prompts** – *i.e. questions to use if discussion is timid, loses steam, dominated by one, too focused on one aspect etc.*
Let's start by talking about how you share your food.<br><br>Can I start with ['sharer' participant UWI]?] Follow prompts.<br><br>Is it the same for you ['family-relative' participant]? Do you also share food? If yes, same prompts. If not, why don't you share food[3]?	With whom do you share it? - with family, friends, neighbours, colleagues, or other?<br><br>Where do you share your meals/ food[4]? This can include places such as your neighbourhood, at university, church, during special occasions, etc.<br><br>How do you do it? - Do you exchange your food for different food from others, give it or receive it as a gift, do something in exchange such as a job, or borrow it?
Let's talk about how often you share your food.	Is it something you do daily, weekly, monthly, only on special occasions, or sporadically?

[3] The recruitment strategy might mainly identify formal sharers but the informal sharing is often taken for granted. The 'family-relative' could cover the less formal practices. And if the 'family-relative' really never shared food, it could still make a really interesting conversation on why not and what are their views on it.

[4] This excludes 'joint meals' within the same household, being that a house and its occupants regarded as a unit.

Can you tell me about the foods you share, or others share with you?<br><br>Can I start with [‘sharer’participant UWI[5]]? Do you share similar foods [‘family-relative’ participant]?	Can you talk about the usual foods you share and consume? These can be foods that you usually get from sharing and can be a major part of your diet<br><br>What about foods that you get through sharing as a treat for yourself or your family? These can be special foods you and your family share and eat occasionally.
Now, you have told me about the foods you share. Can we talk about where this food comes from?	Is it grown in the backyard, sent from relatives abroad, bought in the market, or other?<br><br>Imported from other islands, outside the Caribbean?<br><br>Do you always know it?
Why do you share food? What are the reasons for you and your family to share food?	Issues that could be discussed: availability, price/affordability, diet, awareness of food security, sense of community, social status

What represents for you [‘family-relative’ participant] to share food[6]?<br>And for you [‘sharer’ participant UWI]?	Issues that could be discussed: Class, national, ethnic, gender, religious identities
How does it make you feel?	Can you explain why you are [satisfied or not satisfied] with the food you are sharing? Are you proud of it? Why?
Can we talk about how the food you share is prepared?<br><br>May I start with [‘sharer’ participant UWI]?	Is it a cooked meal or fresh food? Both?<br><br>Who usually does the food preparation or cooking of the food shared? Do you do it together?<br><br>[If cooked meal] What cooking methods do you usually prefer? Fresh/raw, boiled/fried/baked/grilled? Any traditional methods?

[5] Just an indication of facilitating the dyadic interview; order of response might depend on natural flow of conversation and responses

[6] In case the ‘family’-relative’ doesn't share food, it is still interesting to ask what would represent for them to share food. Is this a reason why they don’t share it maybe?

	Is the shared food special in some way as opposed to the 'ordinary food' - not shared, consumed alone?<br><br>Do you also share recipes?
Do you see any differences on these between you two?	How and why do you think it has changed?

Can you think of any moments in your life that changed the way you shared food? How did it change?<br><br>I like to hear first from [‘family-relative’ participant]. And you [‘sharer’ participant UWI]?	For example, you started a garden in your backyard, you moved to a different neighbourhood or town/city, you got married and expanded your family, you started a special diet, etc.
Did COVID-19 have an impact on your food sharing practices? If so, how?	Did you share food more often, less, the same? Why?<br><br>Did you start sharing food with new people or in new places?
Can you think of any other events that had an influence on the way you share food?	For example, climate events (cyclones/hurricanes, droughts, flooding), or changes in food prices, etc.
Have you seen any changes with the food or meals you share? What is different about the foods you share now?	How and why do you think it had changed between generations?

What are the concerns in your community about the environment?	e.g. Climate change, cyclones/hurricanes, droughts, flooding
And about health?	Are obesity and diabetes concerns in your community?
Do you think sharing food can contribute to the environment of your community? If so, how?	Do you think foods shared are produced sustainably?
Do you think sharing food can contribute to the health of your community? If so, how?	Do you think foods shared are healthy? Can contribute to a better diet or food security?

	Do you think the practice of sharing food can contribute to people's wellbeing?
Do you think sharing food can contribute to the control your community has on what people eat?	For example, changing their dependence on what food is available at the market.
Do you think food sharing practices have any influence on the perception that people living in your community have about their actions and consequences? Does it change their feeling of being in control of their lives?	Is this important to you ['sharer' participant UWI]? And for you ['family-relative' participant]?

Closing remarks and thanks

A. Pilot tested topic guide

[15 minutes]
Welcome
Greetings and thank you
Check camera and audio

Introduction of study
[add short summary]

With this study, we want to understand more about how people in the Caribbean share their food and what does it mean for them. Food is a very important part of everyone's life. How we get food and from where we get it can influence our diets and health, our communities the planet, and it can also tell a lot about our culture and traditions.

What we mean by 'Sharing food' is a way of getting food for consumption that people don't pay for it. This can be for example when a friend gives you food from their own backyard garden, or food that you get in return to a small job you did for someone. It can also be food shared during feastings or during activities organized in your community such as cooking together. These are some examples of 'food sharing' but there can be many more! When you 'share food' you can be the one receiving it, or the one giving it or maybe both. The food that it's shared can be fresh foods or cooked meals, canned foods, ready meals, etc. Foods can be shared with family, friends, neighbours or even people you don't know that well. However, 'food sharing' does not include the household meals, and it's not the same than donated food which is food donated by the government or organizations to people in need.

We are a team of researchers at the University of Cambridge with advice from colleagues at the University of West Indies undertaking this study to understand more the Caribbean food culture and how it can help towards a healthier future for the region.

[house rules]: e.g., keeping all contributions confidential, no need for names/real names.

All information we collect during the study will be kept strictly confidential. Any information about you, such as your name and address, will be removed and will be linked only by an ID number so that you cannot be identified from it. We will only use your information for research purposes. We will anonymise your information (using no real names) and may share it with other international researchers, such as the University of West Indies, to support additional research in the future.

Introduction of interview procedure:
approx. 60 minutes [check with participants on time available when scheduling each interview]; semi-structured interview with general areas we would like to cover but meant to be a guide; clarifying that they can put emphasis on particular topics and add topics we haven't covered; explaining that the interview is being tape-recorded and it will be transcribed– but no identifiable information kept.

This interview will last about 1hour. I will ask you general questions about some areas we want to cover but these questions are meant to be a guide. You can put emphasis on particular topics or add topics we haven't talked about if you want to.

The interview will be tape-recorded and transcribed, but neither your name nor any other identifying information will be connected with the audio recording or the transcript.

Individual consent - consent procedure:
(Review participant information sheet and confirm e-sign consent form for both participants)

Did you read and understand the information sheet and consent form? Can I explain anything? Do you have any questions? I would like to stress that we will not identify the specific source of comments in any write ups.

[60 minutes]
Ice breaker - Introductions:
What is your relationship? Can you briefly introduce the other person?

**Main Questions**<br>**Let them talk first before giving prompts.**	**Possible prompts** – *i.e. questions to use if discussion is timid, loses steam, dominated by one, too focused on one aspect etc.*
Let's start by talking about **how you share your food.**<br><br>**Can I start with [student/alumni]?**<br>Follow prompts.<br><br>**Is it the same for you [family-relative]? Do you also share food?**<br>If yes, same prompts.	**With whom do you share it?** - with family, friends, neighbours, colleagues, or other?<br><br>**Where do you share** your meals/ food[8]? This can include places such as your neighbourhood, at university, church, during special occasions, etc.

[8] This excludes 'joint meals' within the same household, being that a house and its occupants regarded as a unit.

If not, why don't you share food[7]?	**How do you do it? -** Do you exchange your food for different food from others, give it or receive it as a gift, do something in exchange such as a job, or borrow it?
Let's talk about **how often you share your food.**	Is it something you do daily, weekly, monthly, only on special occasions, or sporadically?<br><br>Does it change depending on the season?
**Can you tell me about the foods you share, or others share with you**?<br><br>Can I start with [student/alumni [9]]?<br><br>Do you share similar foods [family-relative]?	Can you talk about the **usual foods you share** and consume? These can be foods that you usually get from sharing and can be a major part of your diet<br><br>What about foods that you get through sharing as a treat for yourself or your family? These can be **special foods** you and your family share and eat occasionally.<br>Special occasion and/or food (e.g., food brand)
Now, you have told me about the foods you share. **Can we talk about where this food comes from?**	Is it grown in the backyard, sent from relatives abroad, bought in the market, or other?<br><br>Imported from other islands, outside the Caribbean?<br><br>Do you always know it?
**Why do you share food?** What are **the reasons for you** and your family to share food?	Issues that could be discussed: availability, price/affordability, diet, awareness of food security, sense of community, social status

**What represents for you** [family-relative] to share food[10]?<br><br>And for you [student/alumni]?	Issues that could be discussed: Class, national, ethnic, gender, religious identities

[7] The recruitment strategy might mainly identify formal sharers but the informal sharing is often taken for granted. The 'family-relative' could cover the less formal practices. And if the 'family-relative' really never shared food, it could still make a really interesting conversation on why not and what are their views on it.

[9] Just an indication of facilitating the dyadic interview; order of response might depend on natural flow of conversation and responses

[10] In case the 'family'-relative' doesn't share food, it is still interesting to ask what would represent for them to share food. Is this a reason why they don't share it maybe?

	**How the island you live in affects the way you share food compared to other Caribbean islands?**
**How does it make you feel?**	Can you explain why you are [satisfied or not satisfied] with the food you are sharing? Are you proud of it? Why?<br><br>Are there negative feelings?
Can we talk about **how the food you share is prepared?**<br><br>May I start with [student/alumni]?	Is it a **cooked meal or fresh food**? Both?<br><br>**Who usually does the food preparation** or cooking of the food shared? Do you do it together?<br><br>[If cooked meal] **What cooking methods** do you usually prefer? Fresh/raw, boiled/fried/baked/grilled? Any traditional methods?<br><br>Is the shared food special in some way as opposed to the 'ordinary food' - not shared, consumed alone?<br><br>**Do you also share recipes**?
**Do you see any differences on these between you two?**	How and why do you think it has changed?

Can you think of any **moments in your life that changed the way you shared food?**<br><br>I like to hear first from [family-relative].<br><br>And you [student/alumni]?	For example, you started a garden in your backyard, you moved to a different neighbourhood or town/city, you got married and expanded your family, you started a special diet, etc.<br><br>How did it change?
Did **COVID-19 have an impact** on your food sharing practices? If so, how?	Did you share food more often, less, the same? Why?<br><br>Did you start sharing food with new people or in new places?<br><br>Or new foods?

Can you think of **any other events** that had an influence on the way you share food? Use prompts straight away	For example, climate events (cyclones/hurricanes, droughts, flooding), or changes in food prices, etc.
**Have you seen any changes with the food or meals you share?** What is different about the foods you share now?	How and why do you think it had changed between generations?

**What are the concerns in your community about the environment**?<br><br>Community = people you spend time with, for example.<br>Can have other definitions.	e.g. Climate change, cyclones/hurricanes, droughts, flooding<br><br>Find out what they mean by 'we'
**And about health**?	Are obesity and diabetes concerns in your community?
In what way **sharing food can contribute to the environment of your community**?	Do you think shared foods are produced sustainably? And locally?
In what way **sharing food can contribute to the health of your community**?	Do you think shared foods shared are healthy? Can contribute to a better diet or food security?<br><br>Find out PH knowledge healthy/unhealthy<br><br>Do you think the practice of sharing food can contribute to people's wellbeing?
In what way sharing food can contribute to the **control your community has on what people eat**?<br><br>Only if not mentioned at all before	For example, changing their dependence on what food is available at the market.<br><br>Feel more connected to food?<br><br>Feel more empowered?
**Do you think food sharing practices have any influence on** the perception that people living in your community have about their **actions and consequences**?<br><br>Does it change their feeling of being in control of their lives?<br><br>Only if not mentioned at all before	Is this important to you [student/alumni]?<br><br>And for you [family-relative]?

Closing remarks and thank

Ask for other people interested in participating. Snowball

B. List of eligible Caribbean SIDS

Caribbean SIDS	UWI Open Virtual Campus onsite facilities? (Y/N)
Anguilla	Y
Antigua and Barbuda	Y
Aruba	N
Bahamas	Y
Barbados	Y
Belize	Y
Bermuda	Y
British Virgin Islands	Y
Cayman Islands	Y
Cuba	N
Curaçao	N
Dominica	Y
Dominican Republic	N
Grenada	Y
Guadeloupe	N
Guyana	N
Haiti	N
Jamaica	Y
Martinique	N
Montserrat	Y
Puerto Rico	N
Saint Kitts and Nevis	Y
Saint Lucia	Y
Saint Vincent and the Grenadines	Y
Sint Maarten	N
Suriname	N
Trinidad and Tobago	Y
Turks and Caicos Islands	Y
Unites States Virgin Islands	N

C. Study Advert

Participants needed!
Online research study

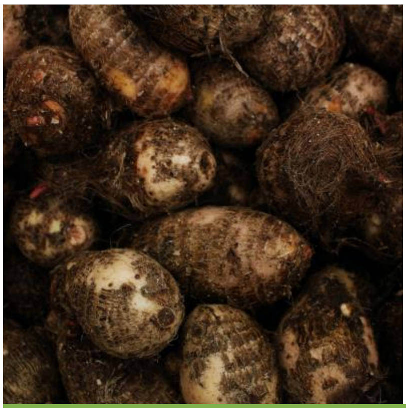
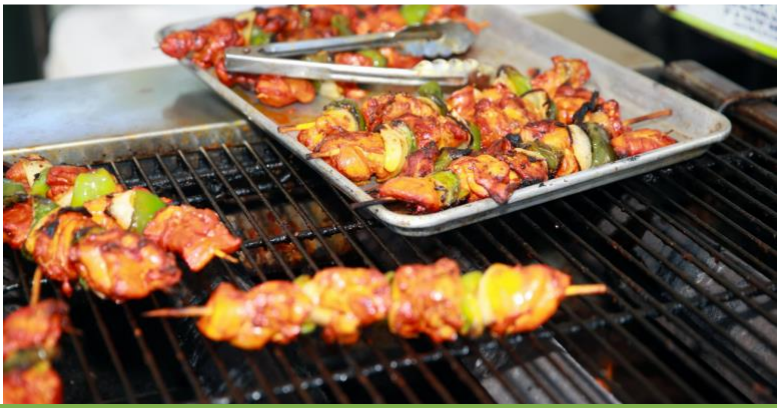

Do you share food with your friends, neighbours, or relatives?

If so, there's a research study you may be interested in participating!

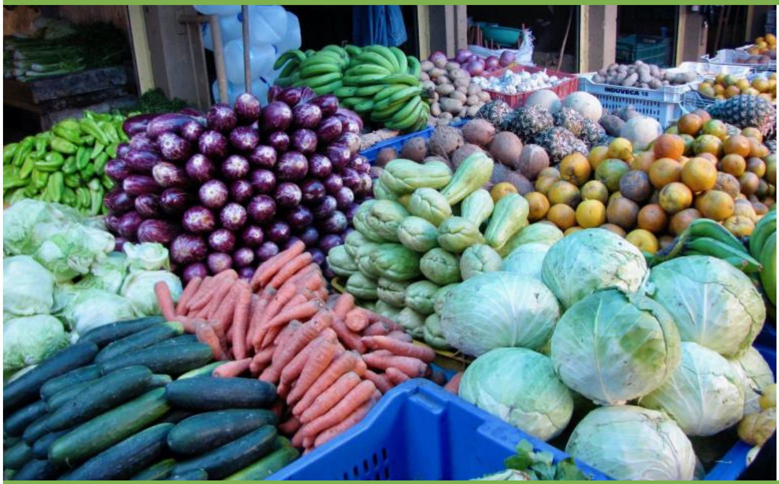
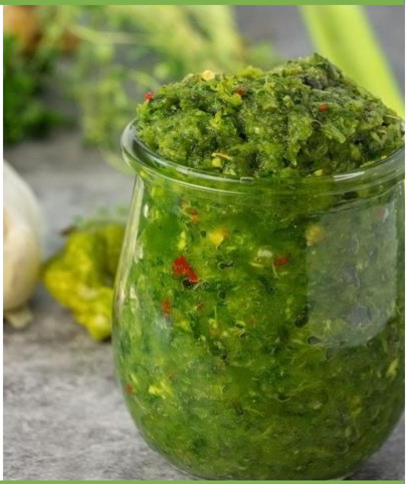

Join a **short interview together with one of your loved ones** and let us know about how you share food. This fun study will take **no more than 1 hour** and you can do it **from your computer at home**

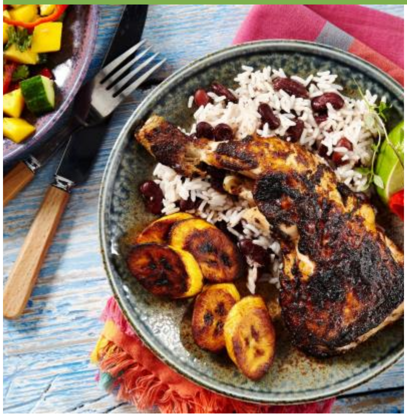

With your participation, you can help to advance research for better food and health in the Caribbean.

Sounds interesting? Visit the link or QR code here to know more about this study. Complete our brief questionnaire and we will be in touch with you!

https://redcap.link/food.sharing.caribbean

THE UNIVERSITY OF THE WEST INDIES
CAVE HILL • MONA • FIVE ISLANDS • OPEN • ST. AUGUSTINE

UNIVERSITY OF CAMBRIDGE

D. Registration of Interest Electronic Form

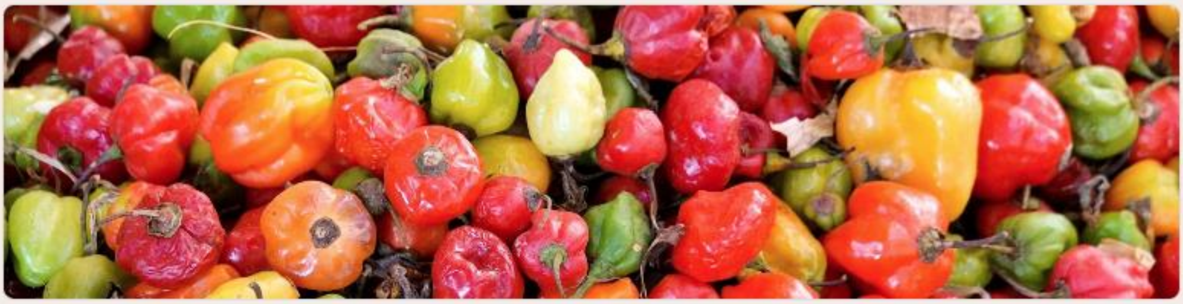

Section 1 of 4

Food Sharing in the Caribbean Islands

Hi!
Thank you for wanting to know a bit more about this study.
Please answer these short questionnaire and we will get in touch with you quickly to give you more info!

What is your age?

Short answer

You must be 18 years or older to participate in this study

Short-answer text

Required

What best represents you? *

- Female
- Male
- Other
- Prefer not to say

Where do you live in the Caribbean? *

This means your usual country of residence. E.g., Trinidad and Tobago

Short-answer text

Section 2 of 4

What do we mean by 'sharing food'?

Description (optional)

'Sharing food' is a way of getting food to eat that people don't pay for it. This can be for example when a friend gives you food from their own backyard garden, or food that you get in return to a small job you did for someone. It can also be food shared during feastings or during activities organized in your community such as cooking together. These are some examples of 'food sharing' but there can be many more! When you 'share food' you can be the one receiving it, or the one giving it or maybe both! The food that it's shared can be fresh foods such as fruits or vegetables, cooked meals, canned foods, ready meals, etc. Foods can be shared with family, friends, neighbors or even people you don't know that well. However, 'food sharing' is not the same than donated food which is food donated by the government or organizations to people in need.

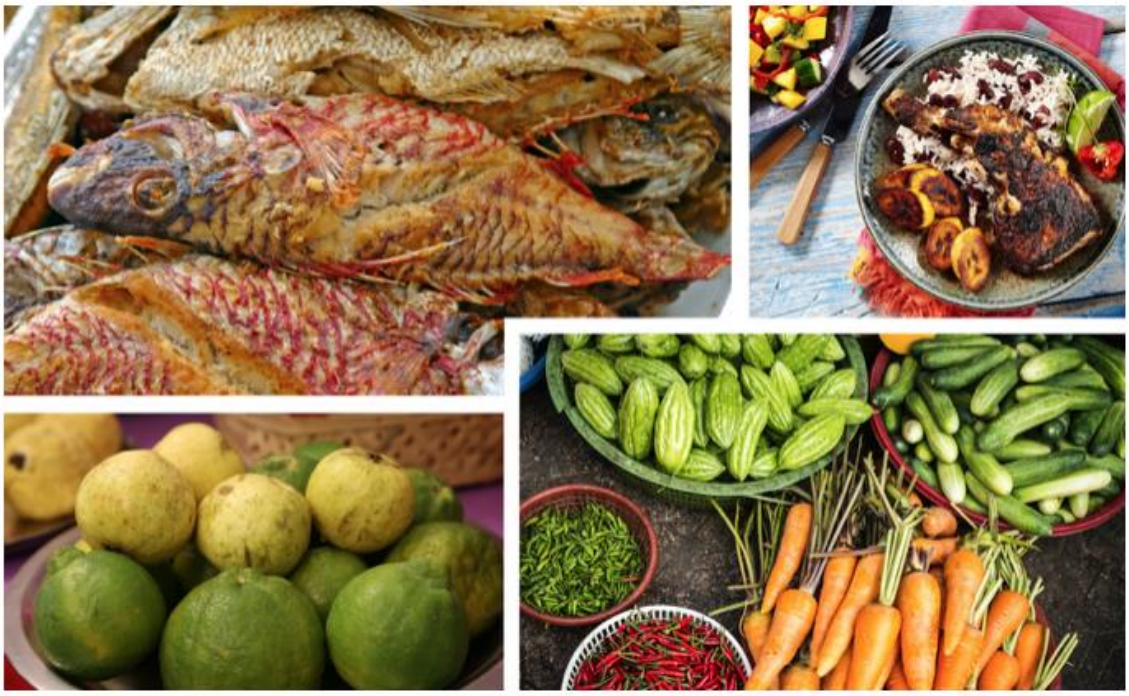

Do you share food with other people at the present? *

Select 'Yes' either if you share food everyday, often, sometimes, or just occasionally.

- ◯ Yes
- ◯ Not at the present, but I used to do it before
- ◯ No, never
- ◯ I'm still not sure what 'food sharing' means!
- ◯ Other...

Section 3 of 4

A virtual interview with a family pair

Description (optional)

In this study, we want to bring you and one of your relatives from another generation, together and have a chat about your experiences sharing food. We will ask you some questions to start an informal chat between you two. It will be done virtually using a videoconferencing platform such as Teams or Zoom.

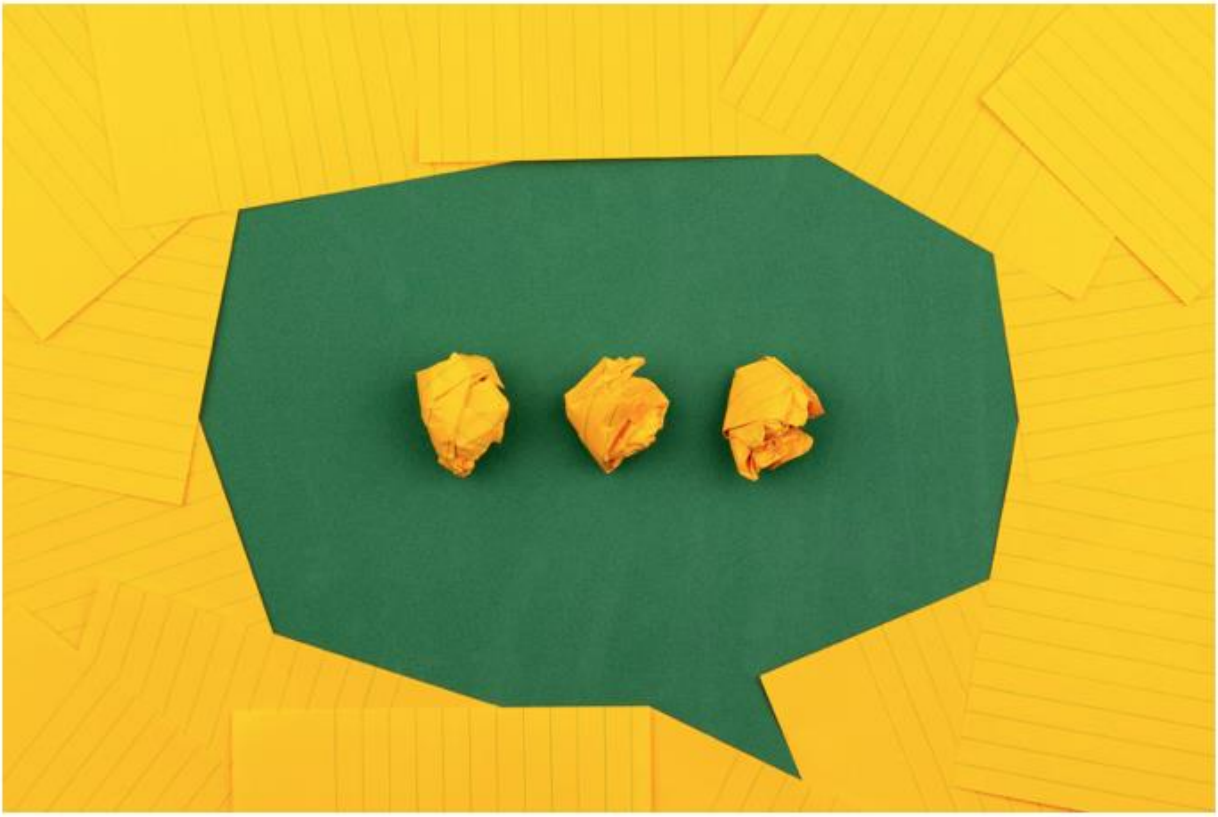

Do you have an electronic device and Internet connection? *

This can be a phone, tablet, laptop, etc. that can have a videoconferencing app such as Zoom, Teams, etc.

- ◯ Yes
- ◯ No
- ◯ Other...

Do you speak English and/or Spanish? *

- Yes
- No
- Other...

After section 3 Continue to next section

Section 4 of 4

Your contact details

Description (optional)

We will get in touch with you to give you with more information about the study, and to answer any questions you may have. Together, we will make sure you have everything you need to take part!

UWI

UNIVERSITY OF CAMBRIDGE

Your name *

Short-answer text

Email *

Short-answer text

E. Participant Information Sheet (PIS)

Participant Information Sheet
Food Sharing in the Caribbean Islands

Summary

1. Please take the time to read the following information carefully. Discuss it with friends and relatives if you wish.
2. Take time to decide whether you wish to take part.
3. If you would no longer like to take part, you can tell us by email at any time.
4. Thank you for reading this.

This study wants to understand more about how people in the Caribbean share their food and what does it mean for them. Food is a very important part of everyone's life. How and from where we get it can influence our diets and health, as well as the wellbeing of our communities and planet. The way we interact with food and the value we place on it can tell a lot about our culture and traditions.

Researchers at the University of Cambridge with advice from colleagues at the University of West Indies will undertake this study to understand more the Caribbean food culture and how it can help towards a healthier future for the region.

You are being asked to participate in this study to share with us how you and another member of your family experience food sharing. Your participation in this study is voluntary.

Contents

1. Why we are doing this study
2. Why am I being asked to take part?
3. What will happen to me if I take part?
4. Possible benefits and disadvantages of taking part
5. More information about taking part
6. Contact for further information

How to contact us

If you have any questions about this study, please talk to:

Research Study Team
food.sharing@mrc-epid.cam.ac.uk

If you have questions about your rights as a person who is taking part in a research study, you may contact:

The Cave Hill Research Ethics Committee

Tel: 417-4847

Email: researchethics@cavehill.uwi.edu

1.Why we are doing this study

What are we studying?

We are studying the way you share food and how this may be changing over time. For example, do you think you share food in the same way your mum or grandad used to do it? Do you maybe see your children sharing food in a different way than you do? We are also interested in knowing if there were any events in your life that influenced your food sharing; for example, did you share food more often with your neighbours during the COVID-19 pandemic? The study also looks at the reasons why you share food. We would love to hear your thoughts about it!

2.Why am I being asked to take part?

We are asking you to take part in this study because you live in a Caribbean Island and you share food with other people, whether that be you have a home garden and share the produce with your neighbours, or you get food from a relative, or you share meals with friends, or you are part of an organisation that does food sharing.

We are especially interested in changes between generations and that's why we ask you to join the study together with a family-relative (older or younger generation than you). We are hoping to include about 15 pairs of participants from different Caribbean Islands.

3.What will happen to me if I take part?

You will firstly be contacted via email by the research study team who will answer any questions you may have. Together, we will confirm that you and your family-relative meet the study eligibility and make sure you have everything needed to take part. For that, we can chat via email or, if you prefer it, we can arrange a virtual call on a date and time of your convenience for a short introduction.

The study will consist of an online Teams or Zoom interview. This means you won't need to leave your home at any time if you don't wish to– you just need to connect to a free video

conferencing platform, as you do in the UWI Open Campus. You and your family-relative will join the interview together and we will have an informal chat for about 60 minutes about how you share food.

4.Possible benefits and disadvantages of taking part

What are the possible disadvantages and risks of taking part?

There are no known disadvantages or risks associated with this study.

What are the possible benefits of taking part?

Whilst there are no immediate benefits for participants of this study, we hope that this work will contribute towards better health and food systems in the Caribbean. With your participation, you can help researchers to understand the Caribbean food culture better and how this can help towards a future more just for all.

5.More information about taking part

Do I have to take part?

No, it is up to you to decide whether to take part in this study. If you or a family member, have an on-going relationship with UWI or University of Cambridge (e.g., as a member of staff, as student or other service user), your decision on whether to take part will not affect this relationship in any way.

Can I change my mind about taking part?

Yes, you can stop participating in the study at any time and without giving a reason.

If I change my mind, what happens to my information?

If you decide to leave the study, we will not collect any more information from or about you.

About the information we have already collected before this point, your rights to access, change or move that information are limited. This is because we need to manage your information in specific ways for the research to be reliable and accurate. More explanation about this is in the Personal Information section below.

Will I receive any payment for taking part?

You will not be paid for taking part in this study.

What if there is a problem?

If you have a concern about any aspect of this study, you should ask to speak to the study research team who will do their best to answer your questions on food.sharing@mrc-epid.cam.ac.uk

If required, independent complaints can be made to the Principal Study Supervisors on louise.foley@mrc-epid.cam.ac.uk or C.Guell@exeter.ac.uk

If you remain unhappy and wish to complain formally, the normal University of Cambridge complaints process is available to you through the University of Cambridge Clinical School Secretary: telephone: +44 (0)1223 333543 or email: schoolsec@medschl.cam.ac.uk
If you have questions about your rights as a person who is taking part in a research study, you may contact the Cave Hill Research Ethics Committee at 417-4847 or by email at researchethics@cavehill.uwi.edu

What will happen to information about me collected during the study?

Information we collect during the study will be kept strictly confidential. Any information about you, such as your name and address, will be removed and will be linked only by an ID number so that you cannot be identified from it. Information will not be used or made available for any purpose other than for research. Anonymised information may also be shared with other international researchers, such as the University of West Indies, to support additional research in the future.

With your permission, information will be processed and stored securely on a secured network drive on computers in the MRC Epidemiology Unit under the data protection regulations at the University of Cambridge in the United Kingdom. General information about how the University uses personal data can be found here https://www.information-compliance.admin.cam.ac.uk/data-protection/research-participant-data.

Codes connecting your individual identity to the stored data records will be kept separately. For accuracy, the study will involve an audio recording. Neither your name nor any other identifying information will be connected with the audio recording or the transcript. The research team will be able to listen to the recordings after which the audiotapes will be transcribed by a trusted and approved external company and the original audio recordings deleted from the recording device.
Transcripts may be reproduced in whole or in part for use in presentations or written products that result from this study.

If you leave the study, we will keep the information about you that we have already obtained. Your rights to access, change or move your information are limited, as we need to manage your information in specific ways for the research to be reliable and accurate. To safeguard your rights, we will use the minimum personally identifiable information possible.

Occasionally our studies may be monitored by our Sponsors. This is to ensure our research is conducted soundly. This procedure is routine and done by fully qualified staff and data confidentiality will always be adhered to. At the end of the study, the confidential records will be stored securely at the MRC Epidemiology Unit for 20 years in accordance with the University of Cambridge data policies and then destroyed.

The University of Cambridge is the sponsor for this study based in the United Kingdom. We will be using information from you in order to do this study and we will act as the data controller for this.

What will happen to the results of the study?

When the study is completed, the results will be reported through publications, research reports and presented at conferences. In all this reporting, you will not be personally identified. This means that the reporting will not include your name or details that will allow others to know that you participated. We will also give you summary of our findings from the study through our newsletters.

Who is organising and funding the study?

This study is organised by the MRC Epidemiology Unit, part of the University of Cambridge, and the University of West Indies. The funders are the Medical Research Council and the Economic and Social Research Council, part of the UK Research and Innovation.

Who has reviewed the study?

This study has been reviewed by an independent group of people, called the Research Ethics Committee, to protect your safety, rights, well-being and dignity. The study has been given a favourable opinion by the UWI Cave Hill Campus Research Ethics Committee and by the Psychology Research Ethics Committee at University of Cambridge.

6.Contact to sign up or get more information

If you want to sign up to participate or have any questions about the study, you can send us an email at food.sharing@mrc-epid.cam.ac.uk or ab2589@mrc-epid.cam.ac.uk and we will be happy to chat with you about what's next!

Research Study Team

Anna Brugulat Panes
Louise Foley
Cornelia Guell
Maddy Murphy
Nigel Unwin
food.sharing@mrc-epid.cam.ac.uk

Principal Investigator

Anna Brugulat Panes
Global Diet and Activity Research Group
MRC Epidemiology, University of Cambridge
ab2589@mrc-epid.cam.ac.uk

Principal Study Supervisors

Louise Foley
Global Diet and Activity Research Group

MRC Epidemiology, University of Cambridge
louise.foley@mrc-epid.cam.ac.uk

Cornelia Guell
The European Centre for Environment & Human Health
University of Exeter
C.Guell@exeter.ac.uk

Local researcher

Madhuvanti (Maddy) Murphy
The University of West Indies
Cave Hill Campus Barbados
madhuvanti.murphy@cavehill.uwi.edu

Thank you for taking the time to consider taking part in this study

F. Consent and Signature

CONSENT AND SIGNATURE

Exploring food sharing practices in Caribbean Small Island States

I confirm that the purpose of the research, the study procedures, and any possible risks or discomforts, as well as the possible benefits that I may receive by taking part in the study, have been explained to me. Alternatives to my taking part have also been discussed and all my questions have been answered. I further confirm that I have been provided with the telephone number(s) of the Principal Investigator for contact in the case of an emergency. I have read this Consent Form and my signature below confirms that I am willing to take part in this study.

Participant's Name (print) ____

Participant's Signature ____

Date ________________________

OPTIONAL:

I am willing to be contacted again in the future about further follow up for this study or any future research.

Yes ☐ No ☐

INVESTIGATOR'S STATEMENT AND SIGNATURE

I have explained the purpose of the research, the study procedures, including those that are investigational, the possible risks and discomforts, and the potential benefits, and have answered all questions regarding the study to the best of my ability. In my opinion, the participant understands these issues and has voluntarily agreed to participate in the study.

Name and Signature of Investigator obtaining Informed Consent:

____

Date ____

A copy of the signed Informed Consent Form must be left with each participant.

If you have further questions about this study, an independent contact from the Research Ethics Committee can be contacted at:

Mrs. Libby Martinez
Administrator
researchethics@open.uwi.edu

G. Transcription Specification Sheet

[Name Study Project]: MRC Epidemiology Unit

Batch: [created by Data Management]
Batch Filename: [created by Data Management]

Data entry instructions:

1. Please be aware that these interviews may contain identifiable information and need to be treated as confidential. Only transcribe what the people say.
2. If the participant is referred to by their name in the interview please only transcribe their initials
3. If any other names are mentioned in the interview please only transcribe the initials
4. Anything in purple needs to be completed

Interview Type	Filename	Voice	Name to be used in transcription	Duration (hh:mm:ss)	Notes	Annex code
A general description of type of interview conducted	*The file name of the audio file being sent for transcription*	*A description of each voices role in the interview*	*What should be used to identify each voice in the transcription*	*The total duration of the audio file*	*Any specific notes for each audio file*	*A reference to any further notes at the end of this document*

H. Letter of local ethics approval

THE UNIVERSITY OF THE WEST INDIES
OPEN CAMPUS
CAMPUS RESEARCH ETHICS COMMITTEE
Tel: (868) 662 2002 ext. 82217; Fax: (868) 645 2424; email: researchethics@open.uwi.edu

---

August 16, 2022

Anna Brugulat Panes
MRC Epidemiology Unit, University of Cambridge Institute of Metabolic Science Box 285 Cambridge Biomedical Campus Hills Road Cambridge CB2 0QQ United Kingdom
Email: ab2589@mrc-epid.cam.ac.uk

Dear Anna Brugulat Panes,

Ref: CREC-OC.0110/07/2022

Title: Exploring food sharing practices in Caribbean Small Island States

I am pleased to advise that your application for research on the above captioned topic has been approved on behalf of the Open Campus Research Ethics Committee.

Approval is specific to the ethical aspect of the proposal and does not imply endorsement of the research methodology or research design. Note that this approval is valid for one (1) year. In the event that you require an extension to this approval, please contact the OC REC Administrator, Libby Martinez, email researchethics@open.uwi.edu for further guidance.

Sincerely,

Dr Emily Dick-Forde
Chair
Campus Research Ethics Committee

Digitally generated by UWIScholar.

I. Letter of Cambridge ethics approval

Milly Bodfish
Secretary

Dr L Foley
MRC Epidemiology Unit
University of Cambridge

UNIVERSITY OF CAMBRIDGE

CAMBRIDGE
PSYCHOLOGY RESEARCH
ETHICS COMMITTEE

10 October 2022

Application No: PRE.2022.087

Dear Dr Foley

Exploring food sharing practices in Caribbean Small Island Developing States: a qualitative study

The Cambridge Psychology Research Ethics Committee has given ethical approval to your research project *Exploring food sharing practices in Caribbean Small Island Developing States: a qualitative study* as set out in your application submitted on 16 September 2022.

The Committee attaches certain standard conditions to all ethical approvals. These are:

(a) that if the staff conducting the research should change, any new staff should read the application submitted to the Committee for ethical approval and this letter (and any subsequent letter concerning this application for ethical approval). An amendment request should also be submitted (guidance at www.bio.cam.ac.uk/psyres/amendments);
(b) that if the procedures used in the research project should change or the project itself should be changed, you should consider whether it is necessary to submit a further application for any modified or additional procedures to be approved;
(c) that if the employment or departmental affiliation of the staff should change, you should notify us of that fact.

Members of the Committee also ask that you inform them should you encounter any unexpected ethical issues.

Ethical approval will expire 31 February 2023. If you require an extension, please submit an amendment request before the expiry date (guidance here: https://www.bio.cam.ac.uk/psyres/amendments).

If you would let us know that you are able to accept these conditions, we will record that you have been given ethical approval.

Yours sincerely

M Bodfish

cc Anna Brugulat Panés
Rebecca Margieson

J. Extended description of Methods.

Research design, participants, and settings

This was an explorative qualitative study to gain understanding of the role of generational, life course and socio-cultural factors in contemporary food sharing practices and experiences in the Caribbean region. We conducted online interviews with intergenerational dyads involved in food sharing in the Caribbean. Using a semi-structured interview guide, we aimed to get insights into the fundamentals of food sharing practices in people's lives, its cultural value, and the factors that shape them over time and across generations. We interviewed 20 participants (10 intergenerational dyads) living in Cayman Islands, Grenada, Jamaica, Saint Lucia, and Trinidad and Tobago between January and March 2023.

We recruited participants through the University of West Indies (UWI) Open Campus. This is the UWI virtual campus with reach across different Caribbean islands offering undergraduate and postgraduate programmes fully taught in English. We chose this platform for recruitment to ensure a broad geographical representation and adequate internet access in this online study. Through this, we also aimed; to reach participants living in geographical areas such as remote islands which would be very costly to visit in-person by the investigators; from diverse socio-economic backgrounds, ages, and gender. E-learning can have significant time, flexibility and economic benefits for students, such as less costs in transport or accommodation; and to reduce the burden of potential barriers to conduct this study such as language barriers, travel restrictions and research-associated costs.

We developed a study advert which was disseminated via email by the UWI Open Campus to all students and alumni to seek expressions of interest. A short electronic form, accessible from the study advert, registered participant's interest and provided an initial screening of eligibility. Communication with interested and potentially eligible individuals followed via email to provide them with the Participant Information Sheet (PIS), answer any remaining questions, confirm eligibility, and help them to identify an intergenerational dyad.

Eligible participants were residents in the Caribbean, 18 years or older, had been involved in food sharing practices, and had an intergenerational partner (familial or other) to participate with in the interview. Generally, we considered participants to be from different generations when they were born over a 15–20-year span, but we allowed shorter spans in three instances to get a better representation. Our study sample included 20 participants (17 female, 3 male) ranging from 18 to 83 years old, and from five Caribbean countries (6 different islands). Four additional participants (2 dyads) who had enrolled in the study dropped out due to unforeseen personal circumstances and time incompatibility with their dyad.

Data collection

We used dyadic interviews as the method to collect the study data. Dyadic qualitative interviewing brings two people, with an existing relationship, together to interact with each other in a conversation guided by open-ended research questions. The method allows each participant to develop personal narratives around a research topic while it stimulates new ideas that might

otherwise be missed. An investigator of this study (A.B-P.) conducted the interviews online from her home based in Cambridge (UK), without the presence of anyone else and using the video teleconferencing software Zoom. In half of the interviews, participants joined from the same location, while in the other half, participants connected from different places within the same island, such as different houses. Each interview lasted between 60 and 80 minutes and followed the pilot tested topic guide. Interviews were recorded using two independent audio recording systems (i.e., Dictaphones) available at the MRC Epidemiology Unit[1], which were tested well in advance. Field notes were taken immediately after each interview by A.B-P. consisting of personal observations and reflections related to it.

Methodological framework and underlying theoretical assumptions

This study employs Reflexive Thematic Analysis (RTA) by Braun and Clarke. A critical approach to language theory is adopted, facilitating the interpretation and attribution of meaning to participant responses, aligning with social constructivism and contextualism epistemologies, as well as relativism ontologies. The study's ontology leans towards relativism, enabling the drawing of conclusions from a small sample size under the assumption that each individual's experience is valid and unique, rejecting a singular truth. Epistemologically, contextualism is favoured, reflecting the framing of food sharing within specific contexts.

Data analysis

Interview transcripts were conducted by an approved third-party transcription company with experience in transcribing Caribbean-English accents, except for one transcript which was transcribed verbatim by A.B-P. due to background noises. Transcripts were not returned to participants for comment and/or correction. We used NVivo Qualitative Data Analysis Software for analysis following a reflexive thematic analysis method which consisted in five main steps: familiarisation with the data, theme and subtheme identification, coding assignation, chart representation and interpretation. Participants did not provided feedback on the findings.
