# Supplementary material for: An exploratory case study of food sharing practices in Caribbean countries through a transition lens using intergenerational dyad interviews
Source: Global Health. 2024 Dec 24;20:88. doi: 10.1186/s12992-024-01094-0 (PMC11669230; doi:10.1186/s12992-024-01094-0)

Additional Table 1. NVivo Codebook

**Name**	**Files**	**References**
'Abundance' in feastings is a good thing	3	6
'Culinary vulnerability' when sharing food	3	6
'Liming' comes with food sharing	3	6
'Modern lifestyle' is responsible for NCDs	2	7
'We can't grow it here'	2	4
A growing-up culture (identity)	9	15
A moral obligation	6	14
Back to 'old times' is needed to progress	2	3
Calorie overconsumption in food sharing	3	5
Climate events affect amount of foods shared	6	12
Close knit allows food sharing	3	6
Community bonding through food sharing	6	18
Community empowerment through food sharing	4	8
Convenience culture can change the palate and affect food sharing	2	6
Convenience food can cause disconnection with what we eat	3	4
COVID-19 affected size of food sharing circles (more limited)	6	10
COVID-19 increased food sharing	1	2
Crime reduces trust to share foods	1	3
Culinary passion drives food sharing	3	5
Diverse and heterogenous cuisines drive food sharing	2	2
Family bonding through food sharing	6	9
Food environment contradicts public health messages	3	8
Food gifts to people in need	5	11
Food import dependence can be reduced with food sharing	3	5
Food imports (in stores) mask local production	1	5
Food offering culture drives food sharing	4	7

**Name**	**Files**	**References**
Food remittances are also shared	1	3
Food sharing in extended families is given for granted	6	12
Shared foods can be unpredictable (seasonality in own grown foods)	5	6
Food sharing adaptation to modern world (social media)	3	5
Food sharing can be with close relationships	7	12
Food sharing can be with strangers	5	8
Food sharing can involve UPFs	4	5
Food sharing can reduce food waste	5	10
Food sharing etiquette and politeness	5	11
Food sharing has non-monetary value	1	2
Food sharing increased food access during COVID-19	2	2
Food sharing is 'a Caribbean thing'	7	13
Food sharing is becoming more organised	2	2
Food sharing is getting commodified	2	4
Food sharing started in times of hardship	4	5
Happiness of helping others (in need) when sharing food	8	12
Heterogenous food sourcing in food sharing	2	3
High food prices (climate change) can enhance food sharing (and growing)	3	4
High food prices (economy) reduce frequency of sharing	4	10
Individualisation reduces food sharing	7	13
Intergenerational differences in cooking shared food	3	5
Intergenerational differences in food sources	1	6
Intuitive or “feel” cooking	7	11
It's not the same if it's not shared (personal satisfaction)	3	6
Little changes between generations in food sharing essence	6	10
Love and care expression through food sharing	9	22
Meals shared are popular Caribbean dishes	4	7
More culinary effort is put when food sharing	7	10

Name	Files	References
Negative impacts of convenience culture, modernisation and urbanisation on food sharing	7	23
Non-monetary economy (work paid with food)	2	3
Own produce (local) is healthier for people and the planet	7	20
Passing family food knowledge when food sharing	6	10
Potential benefit of food sharing in NCDs	6	11
Religious beliefs and gratitude prompt food sharing	6	9
Religious practices promote food sharing	7	18
Risk of young generation losing it	2	3
Seamless food sharing	5	13
Shared Caribbean dishes can be heavy	2	2
Shared drinks are not necessarily healthy	4	7
Shared food comes from the backyard garden	5	12
Shared food is purchased at grocery stores	7	12
Special occasions involve food sharing	7	11
Surplus is key for food sharing to happen	8	19
The younger share more unhealthy stuff	2	5
Time affects behaviour towards convenience culture	4	9
Unexpressed expectation of reciprocity in food sharing	5	7
Younger generations don't do much own food production or wild sources	5	12
Youngers can't share as much as their elders due to nowadays difficulties	3	14

Additional Figure 1. Grouping of codes related to study objective 1.

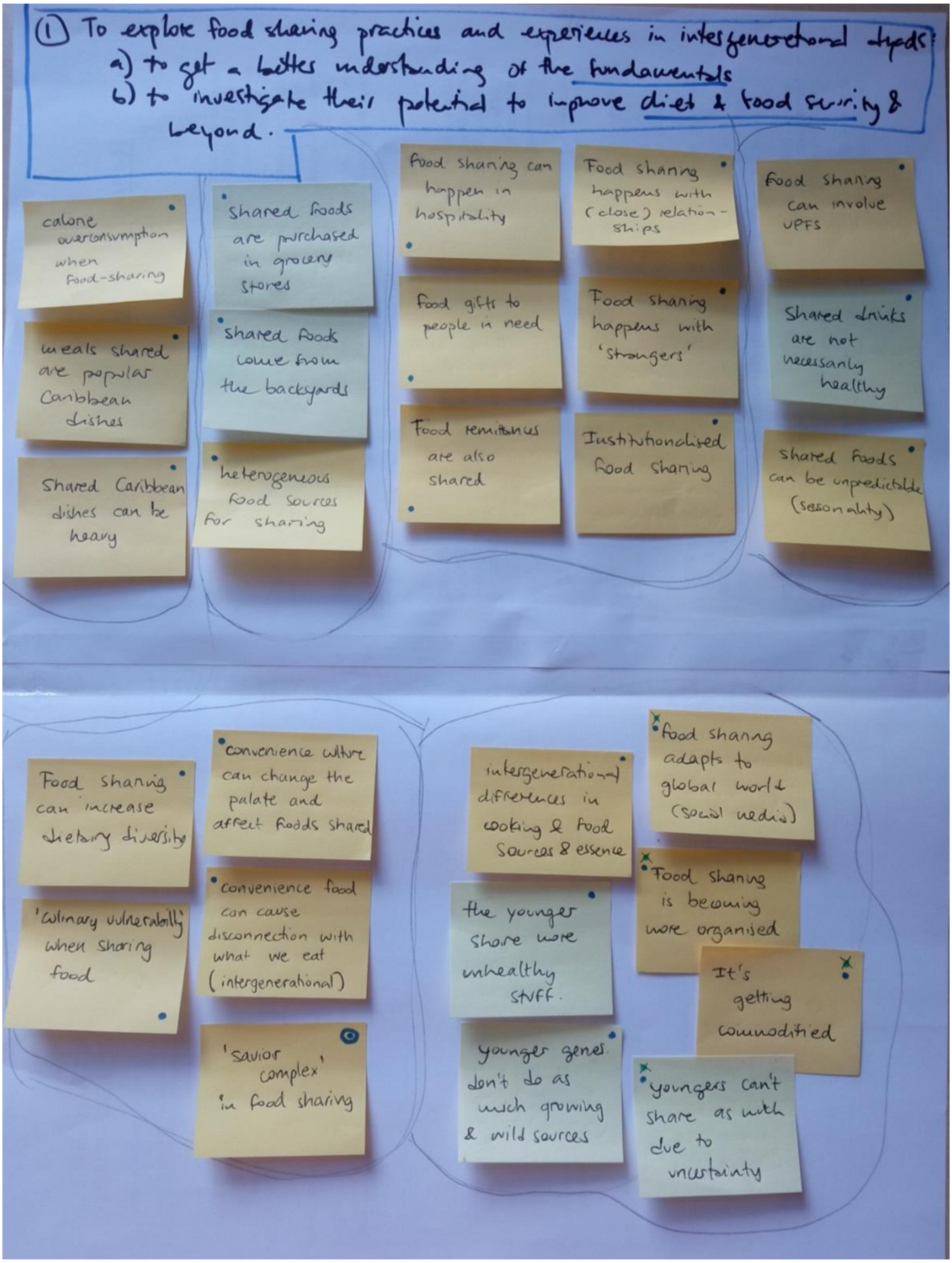

Additional Figure 2. Grouping of codes related to study objective 2.

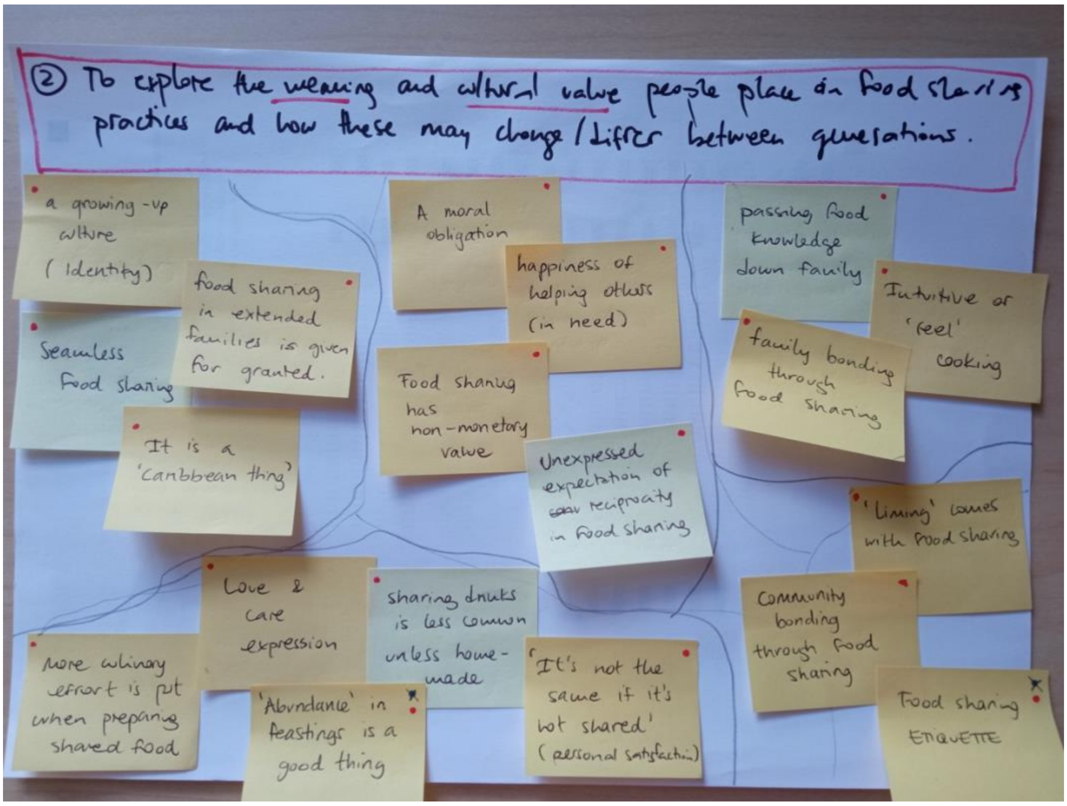

Additional Figure 3. Grouping of codes related to study objective 3.

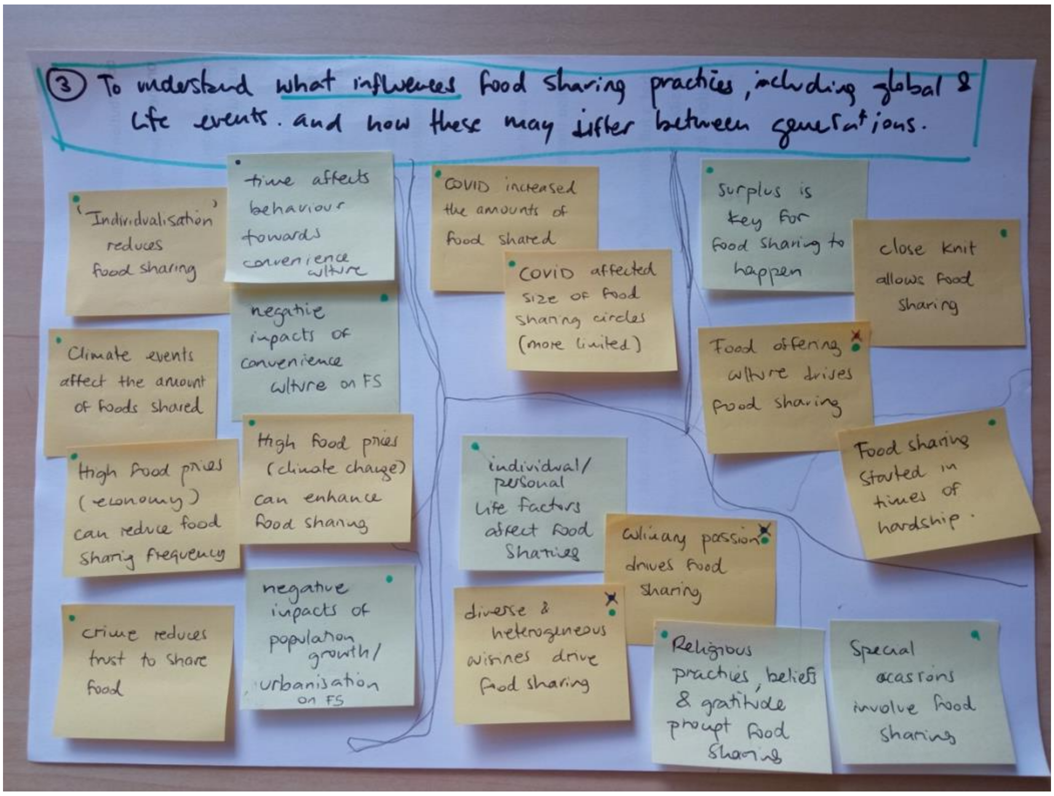

Additional Figure 4. Grouping of codes related to study objective 4.

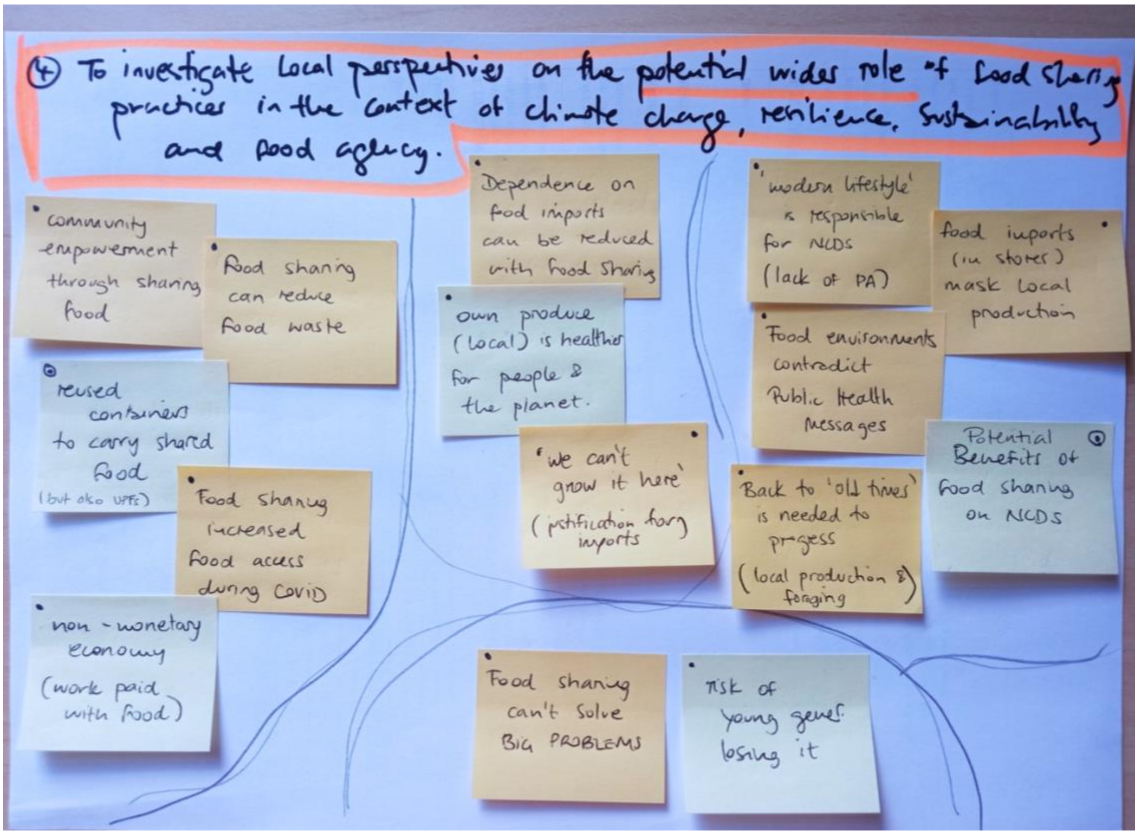

Additional Figure 5. Grouping of codes related to theme 1.

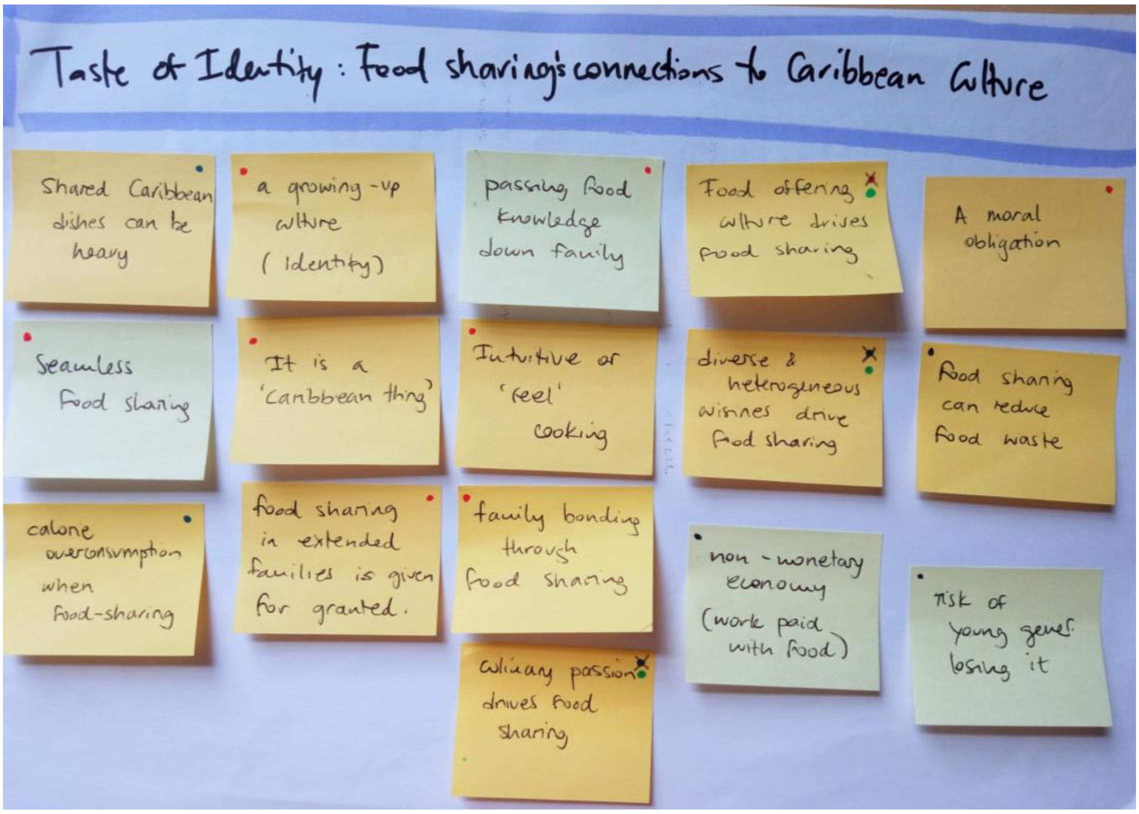

Additional Figure 6. Grouping of codes related to theme 2.

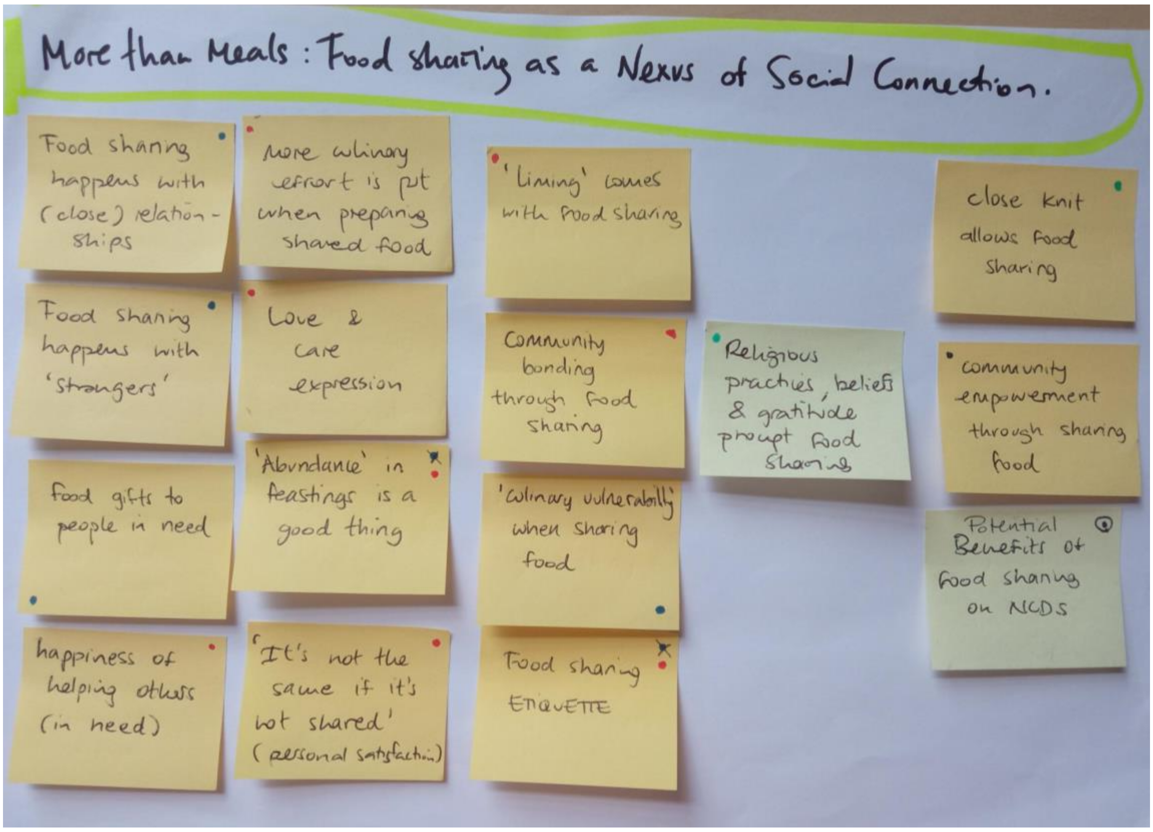

Additional Figure 7. Grouping of codes related to theme 3.

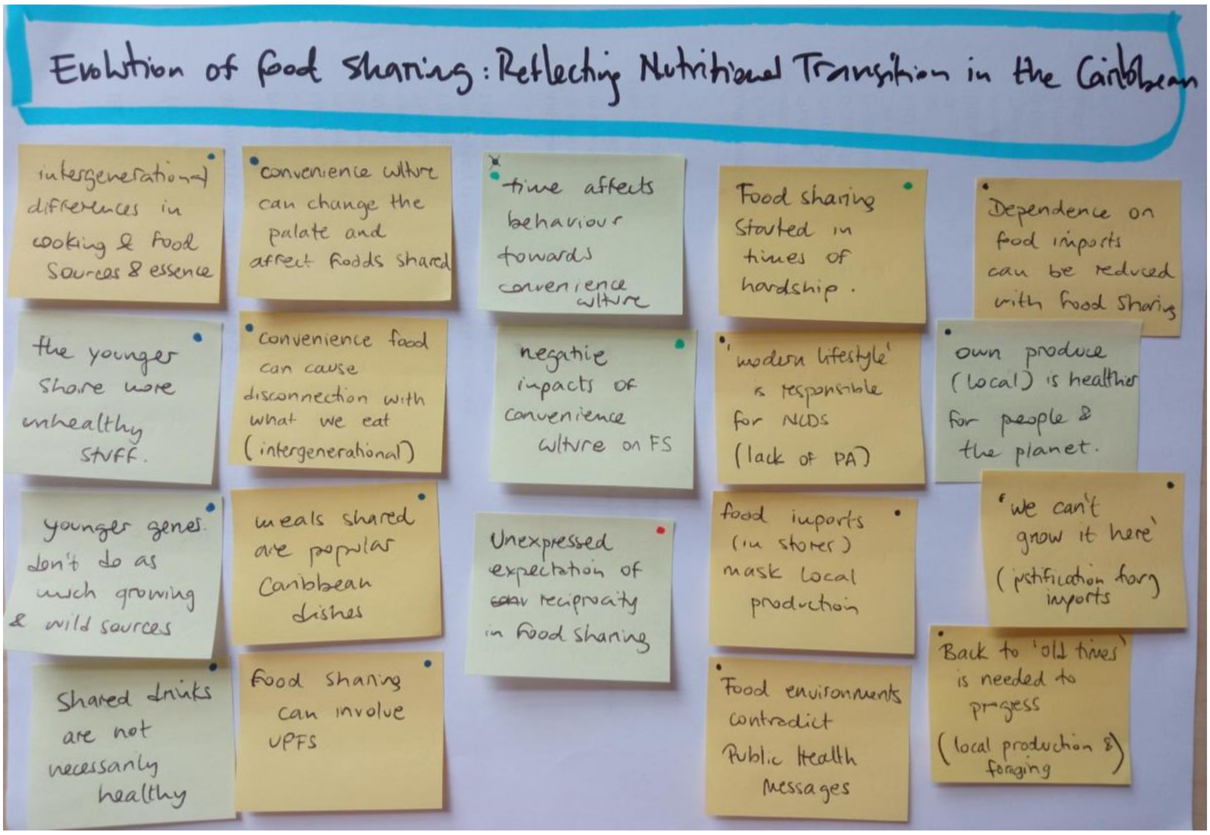

Additional Figure 8. Grouping of codes related to theme 4.

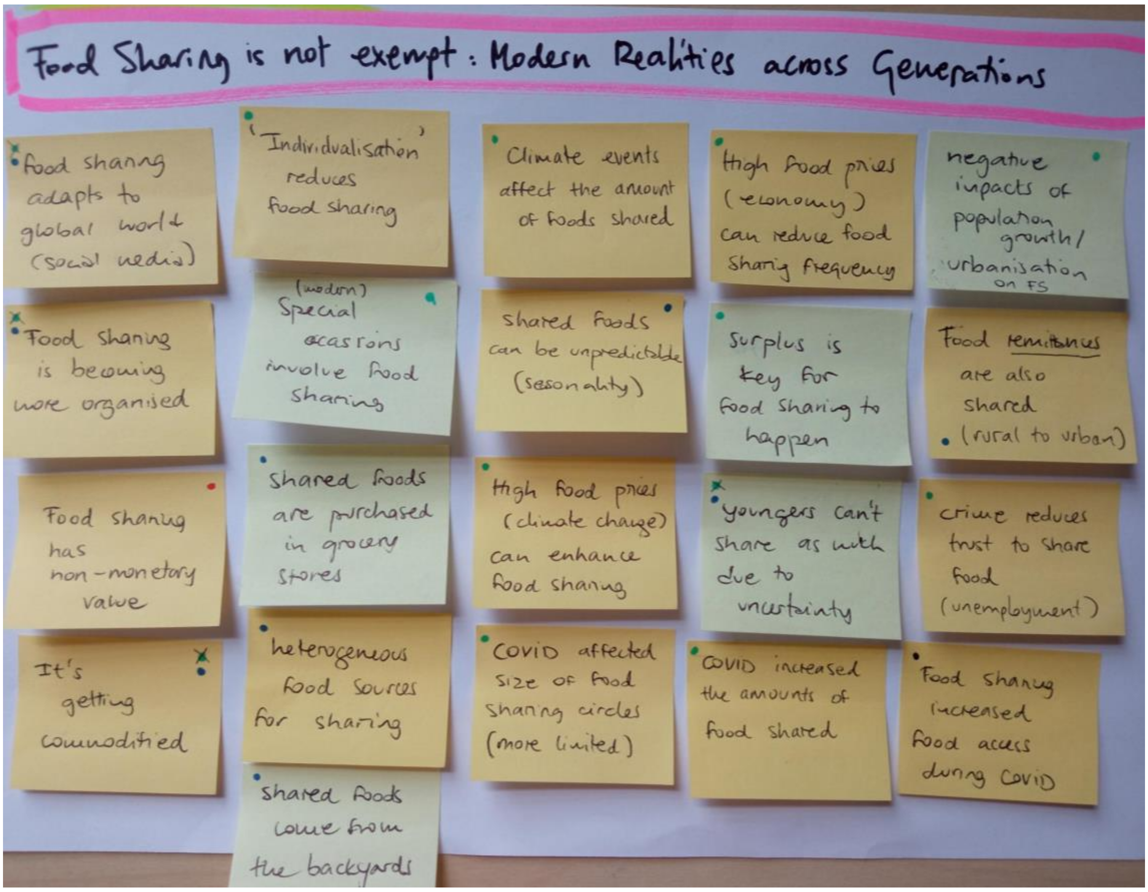

Supplement: Supplementary file 3 — Supplementary Material 3 [file 12992_2024_1094_MOESM3_ESM.pdf]
